# Prediction of relapse-free survival according to adjuvant chemotherapy and regulator of chromosome condensation 2 (RCC2) expression in colorectal cancer

**DOI:** 10.1136/esmoopen-2020-001040

**Published:** 2020-11-20

**Authors:** Christian H Bergsland, Jarle Bruun, Marianne G Guren, Aud Svindland, Merete Bjørnslett, Jørgen Smeby, Merete Hektoen, Matthias Kolberg, Enric Domingo, Teijo Pellinen, Ian Tomlinson, David Kerr, David N Church, Arild Nesbakken, Anita Sveen, Ragnhild A Lothe

**Affiliations:** 1 Department of Molecular Oncology, Institute for Cancer Research, Oslo University Hospital, Oslo, Norway; 2 K.G. Jebsen Colorectal Cancer Research Centre, Clinic for Cancer Medicine, Oslo University Hospital, Oslo, Norway; 3 Institute of Clinical Medicine, Faculty of Medicine, University of Oslo, Oslo, Norway; 4 Department of Oncology, Oslo University Hospital, Oslo, Norway; 5 Department of Oncology, University of Oxford, Oxford, UK; 6 Institute for Molecular Medicine Finland (FIMM), University of Helsinki, Helsinki, Finland; 7 Cancer Research UK Edinburgh Centre, Institute of Genetics & Molecular Medicine, The University of Edinburgh, Edinburgh, UK; 8 Nuffield Division of Clinical Laboratory Sciences, University of Oxford, Oxford, UK; 9 Cancer Genomics and Immunology Group, The Wellcome Centre for Human Genetics, University of Oxford, Oxford, UK; 10 Oxford Cancer Centre, Churchill Hospital, Oxford University Hospitals NHS Foundation Trust, Oxford, UK; 11 Oxford NIHR Comprehensive Biomedical Research Centre, Oxford University Hospitals NHS Foundation Trust, Oxford, UK; 12 Department of Gastrointestinal Surgery, Oslo University Hospital, Oslo, Norway

**Keywords:** RCC2, colorectal cancer, prognosis, immunohistochemistry, chemotherapy

## Abstract

**Background:**

There is a need for improved selection of patients for adjuvant chemotherapy after resection of non-metastatic colorectal cancer (CRC). Regulator of chromosome condensation 2 (RCC2) is a potential prognostic biomarker. We report on the establishment of a robust protocol for RCC2 expression analysis and prognostic tumour biomarker evaluation in patients who did and did not receive adjuvant chemotherapy.

**Materials and methods:**

RCC2 was analysed in 2916 primary CRCs from the QUASAR2 randomised trial and two single-hospital Norwegian series. A new protocol using fluorescent antibody staining and digital image analysis was optimised. Biomarker value for 5-year relapse-free survival was analysed in relation to tumour stage, adjuvant chemotherapy and the molecular markers microsatellite instability, *KRAS*/*BRAF*^V600E^/*TP53* mutations and *CDX2* expression.

**Results:**

Low RCC2 expression was scored in 41% of 2696 evaluable samples. Among patients with stage I–III CRC who had not received adjuvant chemotherapy, low RCC2 expression was an independent marker of inferior 5-year relapse-free survival in multivariable Cox models including clinicopathological factors and molecular markers (HR 1.45, 95% CI 1.09 to 1.94, p=0.012, N=521). RCC2 was not prognostic in patients who had received adjuvant chemotherapy, neither in QUASAR2 nor the pooled Norwegian series. The interaction between RCC2 and adjuvant chemotherapy for prediction of patient outcome was significant in stage III, and strongest among patients with microsatellite stable tumours (p_interaction_=0.028).

**Conclusions:**

Low expression of RCC2 is a biomarker for poor prognosis in patients with stage I–III CRC and seems to be a predictive biomarker for effect of adjuvant chemotherapy.

**Key questions**

**What is already known about this subject?**

- Low expression of regulator of chromosome condensation 2 (RCC2) has been reported to be associated with a poor prognosis in colorectal cancer.
- High expression of RCC2 has been linked to resistance against chemotherapeutics in cancer cell lines.

**What does this study add?**

- The association between low expression of RCC2 and poor patient prognosis is validated in an independent series of colorectal cancer patients, but only in those patients who were not treated with adjuvant chemotherapy.
- A statistically significant interaction was found between RCC2 expression and chemotherapy with respect to relapse-free survival in stage III colorectal cancer.

**How might this impact on clinical practice?**

- If independently validated, expression of RCC2 could be included in risk assessments of non-metastatic colorectal cancer patients; high expression indicating a good prognosis and low expression indicating benefit from adjuvant chemotherapy.

## Introduction

Colorectal cancer (CRC) is a global health challenge with more than 850 000 registered deaths each year.^1^ The management of patients with non-metastatic CRC after surgery of the primary tumour is primarily determined by cancer stage (tumour node metastasis (TNM) system), location and tolerability to chemotherapy.^2^ However, more than half of the patients with stage II and III colon cancer receiving adjuvant chemotherapy are cured by surgery alone, while some relapse after adjuvant chemotherapy, illustrating that current patient selection criteria for adjuvant chemotherapy results in substantial overtreatment and undertreatment.^3^ Molecular profiling has no impact on adjuvant treatment protocols in stage III, while the microsatellite instability (MSI) hypermutator phenotype identifies a low-risk subgroup of patients with no predicted benefit from fluoropyrimidine chemotherapy in stage II.^4^

Several molecular tumour markers in addition to MSI status have been proposed to improve the risk stratification of primary CRCs. Among the most thoroughly investigated markers are *BRAF*^V600E^ and *KRAS* mutations,^5^ and combined analysis of MSI, *KRAS* and *BRAF*^V600E^ improves the prognostic assessment of stage II/III colon cancers, although only modestly when detailed clinicopathological data are included.^6^ Markers of infiltrating non-malignant cells in the tumour microenvironment may have a stronger prognostic potential than the cancer-specific genetic markers.^7^ A prominent stromal component indicates a poor patient survival, while infiltrating lymphocytes are associated with a favourable outcome.^8 9^ The Immunoscore, which is an immunohistochemistry (IHC) -based assay for profiling of tumour-infiltrating CD3^+^/total T-cells and CD8^+^/cytotoxic T-cells, improves the prognostic predictive power over clinicopathological factors alone.^9^ This has prompted development of a TNM-immune-based risk classification for adjuvant treatment decisions, and a recent study of stage III colon cancer patients reported a benefit of 6 months vs 3 months treatment with mFOLFOX6 (fluorouracil, leucovorin and oxaliplatin) in Immunoscore intermediate/high patients, which was not found in Immunoscore low patients.^10^ Of cancer cell-intrinsic markers, the strongest associations with benefit from adjuvant chemotherapy in stage III CRC have been presented for loss of CDX2 expression, although additional supporting data are needed to justify clinical use.^11 12^

Regulator of chromosome condensation 2 (RCC2) was originally identified as a 60 kDa protein in a structure termed the ‘Telophase Disc’ (RCC2 is also known as TD-60).^13^ Functionally, RCC2 is important for proper functioning of the chromosomal passenger complex, which regulates key aspects of mitosis.^14^ Additionally, RCC2 has been shown to be involved in regulation of directional cell migration through its interaction with RAC1.^15^ In recent years, several studies have linked RCC2 to cancer progression,^16–18^ and we have reported that loss of RCC2 expression was associated with a poor prognosis in patients with primary CRC.^19^ Recently, RCC2 has been shown to be under transcriptional regulation by wild-type TP53 and loss of RCC2 in colon cancer cells was found to promote metastasis in an in vivo mouse model.^20^ RCC2 has also been associated with chemotherapy resistance in preclinical cancer models.^17 21^

In this study, we established a robust and accurate protocol for in situ analysis of RCC2 protein expression based on fluorescent IHC using a validated monoclonal antibody. We also investigated the value of RCC2 as a biomarker for prediction of patient outcome in relation to adjuvant chemotherapy in early stage CRC.

## Materials and methods

This study is reported according to the Reporting recommendations for tumor marker prognostic studies (REMARK) (online supplemental table 1).^22^

10.1136/esmoopen-2020-001040.supp1 Supplementary data

### Clinical samples and molecular data

Two independent Norwegian series of primary CRCs (total N=1720) and a British clinical trial series (QUASAR2, N=1196) were analysed (table 1 and online supplemental table 2).

**Table 1:** Clinicopathological characteristics of the three CRC cohorts

	Single-hospital series (consecutive)			Randomised clinical trial
	Norwegian series 1 (1993–2003)	Norwegian series 2 (2003–2012)	P value	QUASAR2 (2005–2010)
Total patients, N	922	798		1196
Age				
Median (range)	73 (29–94)	72 (27–97)	0.60	65 (21–85)
Sex				
Female	485 (53%)	407 (51%)	0.53	507 (42%)
Male	437 (47%)	391 (49%)		689 (58%)
TNM stage				
I	137 (15%)	167 (21%)	0.007	–
II	381 (41%)	288 (36%)		420 (35%)
III	242 (26%)	214 (27%)		776 (65%)
IV	159 (17%)	129 (16%)		–
NA	3	–		–
Resection status				
R0	719 (78%)	651 (82%)	0.09	1196 (100%)
R1	36 (4%)	19 (2%)		–
R2	167 (18%)	128 (16%)		–
Tumour location				
Right colon	365 (40%)	327 (41%)	0.29	445 (42%)
Left colon	301 (33%)	239 (30%)		490 (46%)
Rectum	231 (25%)	218 (27%)		132 (12%)
Synchronous	25 (3%)	14 (2%)		–
NA	–	–		129
MSI status				
MSI	128 (15%)	120 (16%)	0.78	154 (13%)
MSS	712 (85%)	638 (84%)		988 (87%)
NA	82	40		54
*BRAF*^V6ooE^ mutational status				
Wild-type	650 (85%)	288 (83%)	0.66	956 (87%)
Mutated	119 (15%)	57 (17%)		142 (13%)
NA	153	453		98
*KRAS* mutational status				
Wild-type	463 (69%)	238 (69%)	0.94	–
Mutated	204 (31%)	106 (31%)		–
NA	255	454		–
*CDX2* expression status				
Positive	568 (89%)	281 (89%)	0.91	–
Negative	71 (11%)	34 (11%)		–
NA	283	483		–
*TP53* mutational status				
Wild-type	–	145 (42%)	–	–
Mutated	–	202 (58%)		–
NA	–	451		–
RCC2 score, continuous				
median (IQR)	−0.24 (1.06)*	−0.31 (1.08)*	§	5 (3)†
RCC2 score, dichotomised				
RCC2 Low	353 (41%)	309 (41%)	§	439 (40%)
RCC2 High	500 (59%)	444 (59%)		651 (60%)
NA	69	45		106
Adjuvant chemotherapy (in R0-resected stage III patients)‡				
No	165 (74%)	109 (54%)	<0.0001	0 (0%)
Yes	58 (26%)	93 (46%)		1196 (100%)

P values were calculated to determine if there were any statistical differences between the two consecutive Norwegian series; Wilcoxon rank-sum test was used for age, Fisher’s exact test for sex, MSI, *BRAF*, *KRAS*, *CDX2*-status and chemotherapy and χ^2^ test for TNM stage, resection status and tumour location.

*Scaled continuous score.

†Allred score.

‡Adjuvant chemotherapy for stage III CRC was introduced in national guidelines in 1997, and therefore, a lower proportion of patients in Norwegian series 1 received such treatment. Additional details are provided in online supplemental table 2.

§Statistical analysis not relevant since the continuous RCC2 scores were scaled within each series and dichotomisation was performed such that the proportions of RCC2 low/high patients matched previous analysis, as described in the Methods section.

CRC, colorectal cancer; MSI, microsatellite instable; MSS, microsatellite stable; NA, Not available; RCC2, Regulator of chromosome condensation 2; TNM, tumour node metastasis.

Norwegian series 1 (N=922) and 2 (N=798) were two single-hospital series of patients with stage I–IV primary CRCs treated by major surgical resection at Oslo University Hospital in the time periods 1993–2003 and 2003–2012, respectively. The series are population representative for the Southeast of Norway and adjuvant chemotherapy was administered according to national guidelines. Adjuvant chemotherapy has since 1997 been part of standard guidelines for stage III and now also for high-risk stage II colon cancer patients younger than 76 years. This explains why only 58 out of 223 R0-resected stage III patients in the first series (1993–2003) received chemotherapy (table 1 and further details in online supplemental table 2). For patients in the second Norwegian series (2003–2012), adjuvant chemotherapy was given as standard for stage III colon cancer, but on a case-by-case basis according to tolerability in patients above 75 years (6 out of 56 R0-resected stage III colon cancer patients above 75 received). Adjuvant chemotherapy was also administered according to risk assessments in patients with rectal cancer (7 out of 113 R0-resected stage III rectal cancer patients received across the two cohorts). Five-year follow-up for cancer recurrence and survival was complete for all patients except two (one censored at 4.2 years and one with missing information). QUASAR2 was an international, multicentre, phase 3, randomised clinical trial, enrolling 1952 stage III and high-risk stage II CRC patients in the time period 2005–2010.^23^ These patients were randomly assigned to receive capecitabine alone or capecitabine together with bevacizumab in the adjuvant setting.

Construction of tissue microarrays (TMAs) and molecular analyses in the QUASAR2 cohort were performed as previously reported.^23–25^ Patients from QUASAR2 were included in this study based on availability of TMAs and clinical data.

For the Norwegian series 1, MSI-status and *KRAS*- and *BRAF*^V600E^-mutational status have been published.^26^ TMAs with 0.6 mm diameter cores were built from formalin-fixed paraffin-embedded (FFPE) tumour tissue,^19^ and CDX2 expression has been published elsewhere.^12^ For the Norwegian series 2, MSI status, *KRAS*, *BRAF*^V600E^ and *TP53* mutational status, as well as *CDX2*-expression were also previously reported and available for major subsets of the samples (see table 1).^12 26 27^ TMAs were constructed with 1.0 mm diameter cores from FFPE blocks, similarly to the Norwegian series 1. Deficient mismatch repair status was additionally determined by IHC for the mismatch repair proteins MLH1 (MutL homolog 1), MSH2 (MutS homolog 2), MSH6 (MutS homolog 6) and PMS2 (PMS1 homolog 2) in the Norwegian series 2 (online supplemental methods).

Individual patient data cannot be shared.

### Procedures

Two procedures for IHC-based analyses of cytoplasmic RCC2 expression were used. Norwegian series 1 has previously been analysed for nuclear and cytoplasmic expression using a standard DAB (3,3'-Diaminobenzidine)-based chromogenic protocol and a polyclonal antibody, combined with manual scoring of RCC2 staining by the Allred method, providing eight categories of RCC2 expression based on staining proportion and intensity.^19^ The same protocol was here used for analysis of the QUASAR2 trial cohort (details in online supplemental methods). Samples with a cytoplasmic Allred score <5 were scored as having low expression of RCC2. Further, we developed a new protocol for scoring RCC2 expression, based on a monoclonal antibody against RCC2, fluorescent staining and digital image analysis by applying the Vectra 3 Imaging platform and Inform analysis software (Akoya Biosciences) (online supplemental methods). This method was technically validated on the Norwegian series 1, and then used to analyse the Norwegian series 2. With this new protocol, both cytoplasmic and nuclear expressions of RCC2 were scored. The fluorescence protocol provided continuous data for RCC2 expression, and the threshold for dichotomisation into high and low expression groups was set to match the percentage of tumours in each category in our previous analysis of Norwegian series 1 with the chromogenic protocol (41st percentile).^19^ This was also in line with the percentage of samples in each category in the QUASAR2 cohort (40.3% scored into the low RCC2 category with an Allred score <5). Totally 2916 CRCs from the three patient series were analysed for RCC2 expression, and 2696 (92.5%) tumours were evaluable. Online supplemental figure 1 shows an overview of patient samples included in the main analyses, and online supplemental figure 2 shows an overview of the methodology applied to each cohort.

### Statistical analyses

All statistical analyses were performed in RStudio V.1.1.383 with R V.3.6.1. All evaluable samples were used for association and correlation analyses (online supplemental figure 1). Correlations between RCC2 and clinicopathological and molecular variables were tested using the functions fisher.test, wilcox.test, kruskal.test, chisq.test and cor, depending on the nature of the variables (specified in the relevant tables). The continuous RCC2 score was used for correlation analyses where applicable. The outcome evaluated was 5-year relapse-free survival (RFS) and was defined according to guidelines by Punt *et al*; time from surgery to death from any cause, or to recurrence.^28^ (Although disease-free survival (DFS) was used as endpoint in the original publication of the QUASAR2 trial data,^23^ this definition of DFS corresponds to RFS in the guidelines by Punt *et al*,^28^ as information on second primary cancer events were not available). Only stage I–III patients with no residual tumour and a resection margin >1 mm (R0-resection) were included in survival analyses (online supplemental figure 1). Survival analyses were performed using the survival package (V.2.43–3). Kaplan-Meier survival curves were compared using the log-rank test. HRs and 95% CIs were estimated by univariable and multivariable Cox proportional hazards models. Covariables included in the multivariable model for patients with stage I–III CRC and who did not receive chemotherapy were based on clinical relevance, and also included relevant molecular variables, which are listed in table 1 (*TP53* mutational status was not included in the model due to a low proportion of samples with available *TP53* data). A full model including all variables was evaluated and only patients with complete data for all variables were included in the analysis. Since a large proportion of samples in the Norwegian series 2 were missing data for *KRAS*, *BRAF*^V600E^ and *CDX2*, a multivariable model excluding these covariables was also calculated. The two consecutive Norwegian series were pooled where relevant to increase the number of complete observations in statistical analyses. The multivariable analyses were stratified by cohort (Norwegian series 1 vs 2) since they were collected over different time periods and the Norwegian series 2 had a 5-year RFS of 64% compared with 58% in the Norwegian series 1 (online supplemental figure 3). The only statistically significant differences between these two series with respect to patient characteristics was a somewhat higher proportion of stage I and a lower proportion of stage II in the Norwegian series 2 compared with 1, and that a higher proportion of patients received adjuvant chemotherapy in the Norwegian series 2 compared with 1 (table 1). This is explained by the fact that national guidelines were updated in 1997 (described above). Therefore, analyses of the association between RCC2, adjuvant chemotherapy and survival were also performed within each series individually (online supplemental material). Survival analyses in the Norwegian series were performed with RCC2 both as a dichotomised and as a continuous variable, and results from analyses of the continuous expression data are reported for all main models in online supplemental table 7. The Wald statistic was used to estimate the p value for continuous RCC2 scores and for the effects of individual variables in multivariable models. Testing of proportional hazards assumptions were performed with the cox.zph function in the survival package. The assumption of proportional hazards was met in all analyses, except for in univariable survival analysis of age, TNM stage and *CDX2* expression, as indicated in table 2, and the multivariable model in online supplemental table 6. This model was therefore also evaluated on stratification by TNM stage and age, which gave similar results for the prognostic value of RCC2 (data not included). The survMisc package was used to calculate the relative proportion of explained variation in 5-year RFS by each variable included in multivariable analysis.^29^ Kaplan-Meier plots were generated using the survminer package (V.0.4.3). All statistical tests were two sided and p values <0.05 were considered significant. Bonferroni correction was used to account for multiple testing; subgroup analyses of RCC2 according to treatment with chemotherapy (including stratified analysis by MSI-status) and according to *TP53* status were adjusted by a factor of 8, and are reported in the relevant figures.

**Table 2:** Univariable and multivariable 5-year relapse-free survival analyses in stage I–III chemotherapy untreated patients in the pooled Norwegian series

Variable	Strata	Patients, N	Univariable analysis		Multivariable analysis N=521, events=201	
			HR (95 % CI)	P value	HR (95 % CI)	P value
RCC2	High	691	1	–	1	–
	Low	426	1.54 (1.28 to 1.86)	<0.0001	1.45 (1.09 to 1.94)	0.012
Sex	Female	620	1	–	1	–
	Male	577	1.09 (0.91 to 1.30)	0.37	1.12 (0.84 to 1.50)	0.43
Age	Below median†	520	1	–	1	–
	Above or equal to median†	677	1.83 (1.51 to 2.22)	<0.0001*	1.85 (1.35 to 2.54)	0.0001
TNM stage	I	302	1	–	1	–
	II	622	1.71 (1.32 to 2.21)	<0.0001*	1.34 (0.90 to 2.02)	0.15
	III	273	3.12 (2.37 to 4.10)	<0.0001*	2.56 (1.70 to 3.86)	<0.0001
Tumour location	Right colon	469	1	–	1	–
	Left colon	332	1.19 (0.96 to 1.49)	0.12	1.33 (0.92 to 1.92)	0.13
	Rectum	367	0.99 (0.79 to 1.24)	0.95	1.16 (0.77 to 1.73)	0.48
	Synchronous	29	1.19 (0.66 to 2.14)	0.56	0.83 (0.30 to 2.30)	0.71
MSI status	MSI	198	1	–	1	–
	MSS	911	1.28 (0.99 to 1.67)	0.059	2.47 (1.32 to 4.63)	0.0047
*BRAF*^V600E^ mutational status	Wild-type	652	1	–	1	–
	Mutated	124	0.95 (0.69 to 1.30)	0.75	1.72 (1.00 to 2.96)	0.051
*KRAS* mutational status	Wild-type	490	1	–	1	–
	Mutated	209	1.22 (0.95 to 1.56)	0.13	1.07 (0.77 to 1.47)	0.70
*CDX2* expression status	Positive	610	1	–	1	–
	Negative	68	1.22 (0.83 to 1.79)	0.31*	1.86 (1.15 to 3.02)	0.012

Multivariable analysis was stratified by series.

*Violates proportional hazards assumption in univariable analysis.

†Above/below median age (73) of all patients in the pooled series.

MSI, microsatellite instable; MSS, microsatellite stable; RCC2, Regulator of chromosome condensation 2; TNM, tumour node metastasis.

## Results

### Robust method for fluorescent IHC-based analysis of RCC2 expression

**Figure 1:**
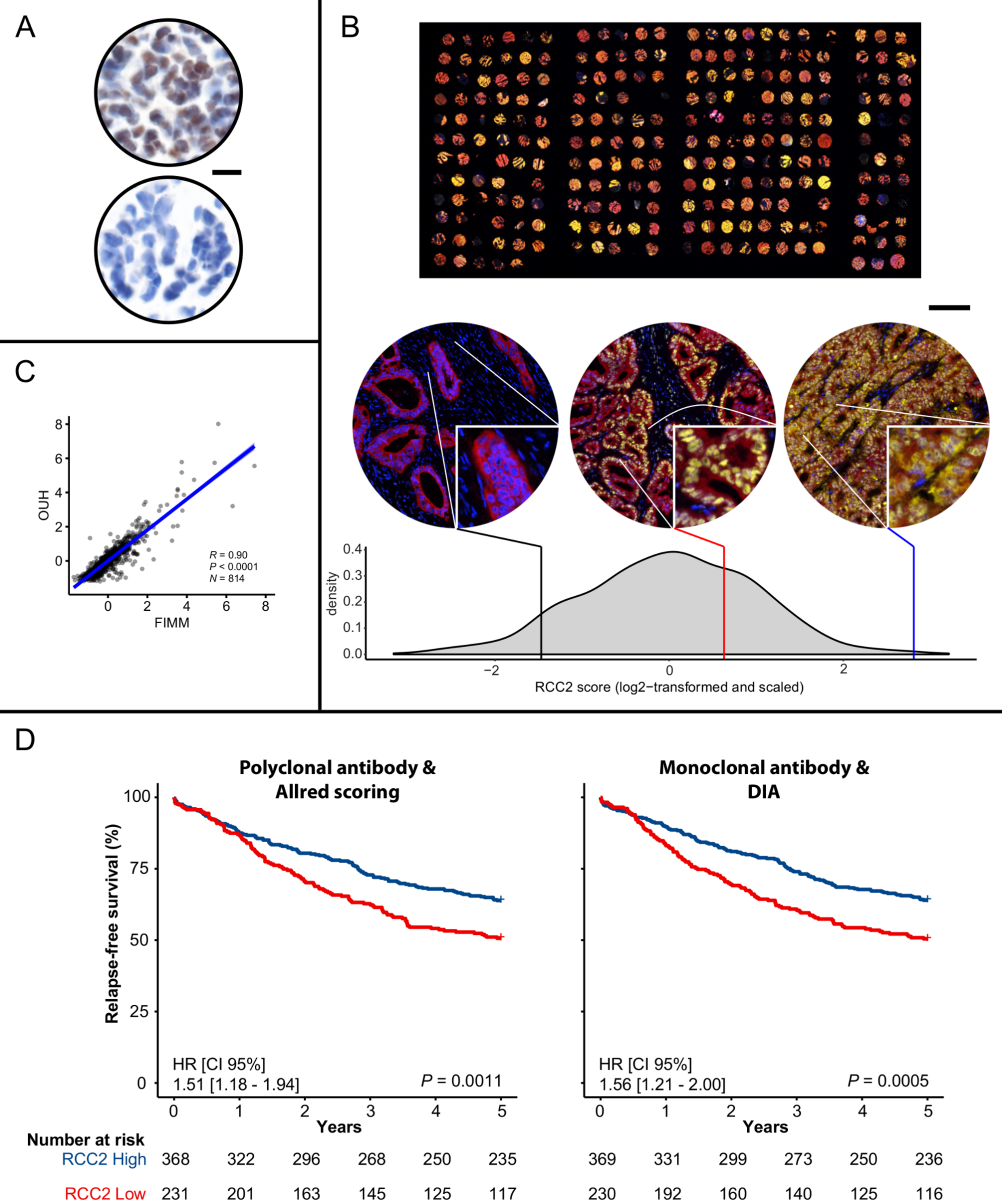
Methodological development and technical validation of in situ protein expression analysis of RCC2 in the Norwegian series 1. (A) HAP1 wild-type cells showing staining (top) and HAP1 *RCC2* knockout cells showing absence of staining (bottom) against RCC2. Scale bar equals 10 µm (×40). (B) Top: one of the TMAs in the Norwegian series 1; RCC2 is shown in yellow, epithelial/tumour tissue in red and DAPI in blue. Scale bar equals 2 mm. Bottom left: a sample demonstrating absence of immunoreactivity against RCC2. Middle: a sample with predominant nuclear localisation of RCC2, having also some in the cytoplasm. Right: a sample showing strong staining against RCC2 in both nuclear and cytoplasmic cell compartments. Where on the scale of the continuous cytoplasmic RCC2 scores these samples are located is shown on the density plot below. Scale bar equals 100 µm in tumour core images (lower right portion of each image is ×3 zoomed). (C) Comparison of cytoplasmic RCC2-scoring at two different institutions using different digital image analysis platforms (x-axis; scores obtained at FIMM, y-axis; scores obtained at OUH, details in online supplemental methods). Correlation coefficient was calculated by Pearson’s method. (D) The association between RCC2 and 5-year relapse-free survival in stage I–III patients was assessed using the original method for scoring RCC2 (polyclonal antibody, chromogenic staining and Allred scoring through visual analysis, data previously published in ref. 19; left panel), and using the new method for scoring RCC2 (monoclonal antibody, fluorescent staining and digital image analysis; right panel). Only samples scored by both methods were included in the comparison. DAPI, 4',6-diamidino-2-phenylindole; DIA, digital image analysis; FIMM, Institute for Molecular Medicine Finland; OUH, Oslo University Hospital; RCC2, Regulator of chromosome condensation 2.

We implemented a new protocol for evaluation of in situ RCC2 protein expression on a continuous scale, based on fluorescent IHC staining. The protocol was found to be robust in Norwegian series 1 (figure 1A–D). First, specificity of the monoclonal antibody against RCC2 was confirmed in an HAP1 RCC2 knockout cell-line model (figure 1A). Second, RCC2 showed a varied nuclear and cytoplasmic staining pattern, with a large range of expression values (figure 1B). Third, fluorescence analyses of RCC2 expression using two different digital pathology solutions were concordant (Pearson’s r=0.90, figure 1C, described in online supplemental methods). Cytoplasmic and nuclear expression of RCC2 were correlated among tumours (Pearson’s r=0.77); however, cytoplasmic RCC2 staining was more strongly associated with patient outcome (online supplemental figure 4). This was in line with previous results,^19^ and all further analyses are therefore based on the cytoplasmic expression of RCC2. Importantly, the association between cytoplasmic RCC2 and 5-year RFS among stage I–III patients was similar when compared with our previously published results from DAB-based visual scoring of RCC2 in this patient series (DAB-based visual: HR=1.51, p=0.0011; fluorescence-based digital: HR=1.56, p=0.0005; N=599, figure 1D).^19^ We further compared methodologies by association analyses with molecular variables (online supplemental table 3). There were no significant associations between RCC2 expression and *KRAS* mutations, *BRAF*^V600E^ mutations or *CDX2* expression by either method. Low RCC2 expression was more frequently found in microsatellite stable (MSS) tumours, although this association was not significant by the new RCC2 scoring method with monoclonal antibody and digital analysis (p=0.06).

### Low RCC2 expression is associated with advanced cancer stage

Totally 2696 CRCs were scored for RCC2 expression across the two Norwegian series and QUASAR2 (92.5% of the analysed samples, online supplemental figure 1). Of these, 1101 (41%) were scored with low RCC2 expression (details in methods). Low RCC2 expression was associated with higher cancer stage in all three cohorts (p<0.001, online supplemental tables 4 and 5), but not with any other clinicopathological factors (patient age, sex or tumour location) or molecular markers (*KRAS*, *BRAF*^V600E^, *TP53* mutation status or *CDX2* expression). A significant association with MSI status was found only in Norwegian series 2 (p=0.0042). Of note, RCC2 expression was not different between patients who did and did not receive adjuvant chemotherapy for stage III CRC in the two Norwegian series (online supplemental table 4), or between the two adjuvant treatment groups in QUASAR2 (online supplemental table 5).

### Low expression of RCC2 is associated with benefit from chemotherapy in stage III CRC

**Figure 2:**
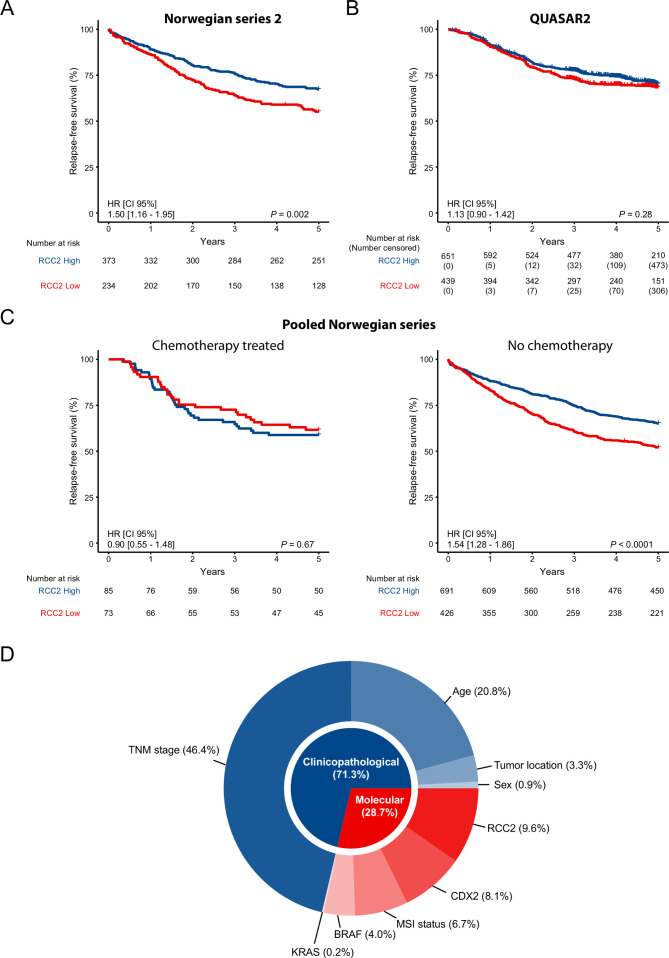
Five-year survival according to RCC2 status in stage I–III patients of the Norwegian series 2 (A), the QUASAR2 cohort (B), and in the pooled Norwegian series split by adjuvant chemotherapy (C). The relative proportion of explained variation in 5-year relapse-free survival for each clinicopathological and molecular variable in multivariable analysis of stage I–III CRC patients who did not receive adjuvant chemotherapy was calculated (D). CRC, colorectal cancer; MSI, microsatellite instability; RCC2, Regulator of chromosome condensation 2; TNM, tumour node metastasis.

**Figure 3:**
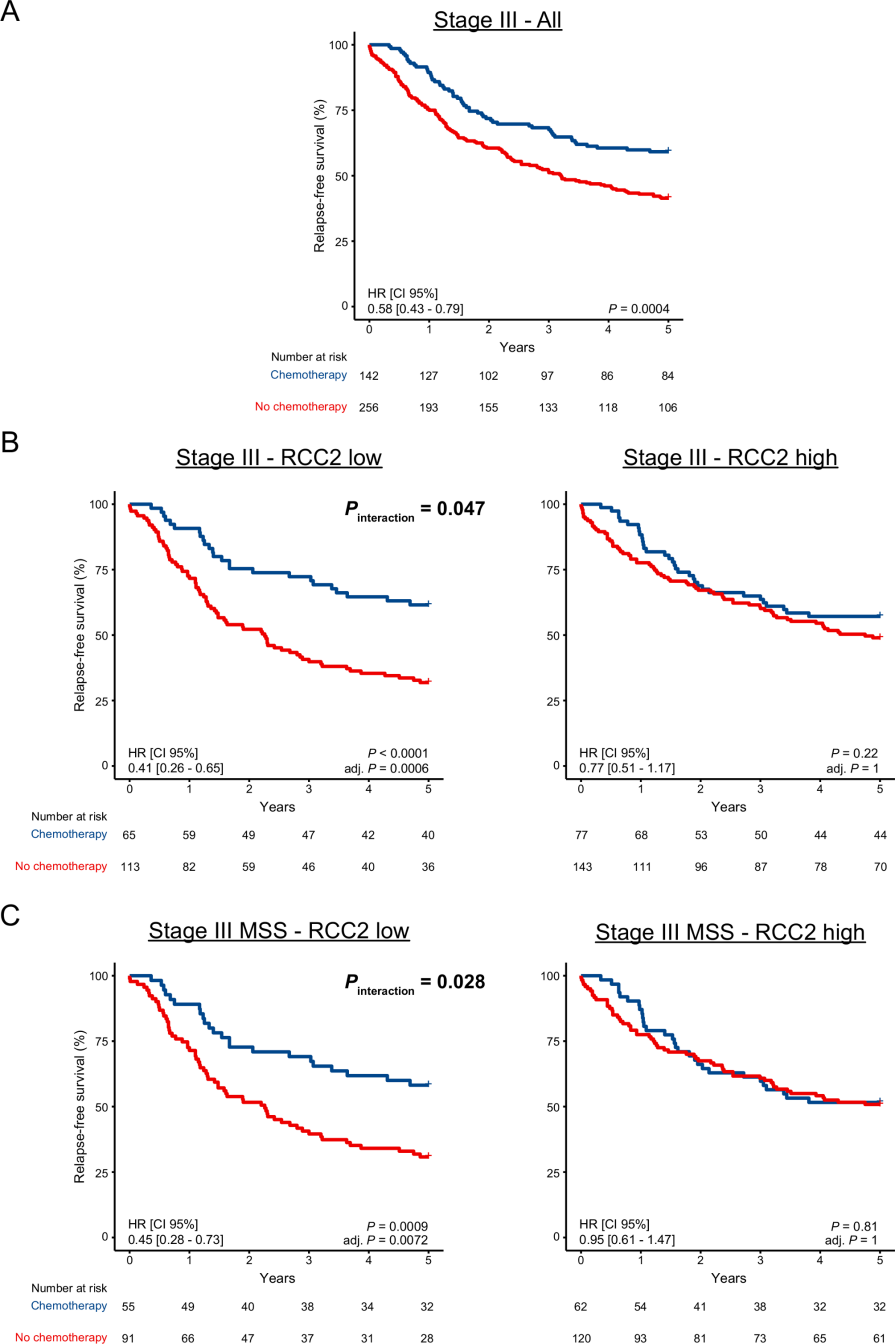
Low RCC2 is associated with benefit from chemotherapy in stage III patients of the pooled Norwegian series. Benefit of adjuvant chemotherapy was assessed in all stage III patients (A), according to low/high RCC2 (B) and according to low/high RCC2 in MSS patients only (C). MSS, microsatellite stable; RCC2, Regulator of chromosome condensation 2.

Analysis of stage I–III patients in the independent Norwegian series 2 validated the prognostic value of low RCC2 expression for 5-year RFS (HR 1.50, 95% CI 1.16 to 1.95, log-rank p=0.002, N=607, figure 2A). However, there was no significant difference in the 5-year RFS rate according to RCC2 expression in the QUASAR2 cohort (HR 1.13, 95% CI 0.90 to 1.42, log-rank p=0.28, N=1090, figure 2B), irrespective of cancer stage (stage II or III, online supplemental figure 5) or treatment regimen (online supplemental figure 6). As all patients in QUASAR2 received adjuvant chemotherapy, and this was the case for only 9% and 16% of stage I–III CRCs in the Norwegian series 1 and 2, respectively (online supplemental table 2), we speculated that this discrepancy could be due to an interaction between RCC2 and chemotherapy. The two Norwegian series were pooled for further analyses, and this confirmed that RCC2 was not prognostic in the subgroup of patients who did receive adjuvant chemotherapy (HR 0.90, 95% CI 0.55 to 1.48, log-rank p=0.67, N=158, figure 2C), irrespective of the treatment type (online supplemental figure 7). In contrast, low expression of RCC2 was significantly associated with worse outcome among patients who did not receive chemotherapy (HR 1.54, 95% CI 1.28 to 1.86, log-rank p<0.0001, N=1117, figure 2C and online supplemental figure 8). A test for interaction in stage III patients showed that adjuvant chemotherapy was associated with higher 5-year RFS rate in patients with low RCC2 expression (p_interaction_=0.047, figure 3A, B and online supplemental figure 9). Stratification according to MSI status further showed that low expression of RCC2 was associated with benefit from chemotherapy only in the MSS subgroup (p_interaction_=0.028, figure 3C and online supplemental figure 10). Analyses excluding rectal cancer patients showed the same trend as when all patients were analysed together (online supplemental figures 11 and 12). Exploratory analyses suggested that the benefit from chemotherapy in the RCC2-low subgroup was independent of *CDX2* expression, but the number of tumours with low *CDX2* expression was too small to draw a firm conclusion (online supplemental figure 13).

### Prognostic value of RCC2 is independent of clinicopathological and molecular markers in patients with no adjuvant treatment

The prognostic value of RCC2 among patients in the pooled Norwegian series who did not receive adjuvant chemotherapy was further analysed in a full multivariable Cox model including other molecular and clinicopathological parameters. RCC2 retained independent prognostic value in this model (HR 1.45, 95% CI 1.09 to 1.94, p=0.012, N=521, table 2). A model excluding *KRAS*, *BRAF*^V600E^ and *CDX2* as covariables allowed for inclusion of more patients and gave similar results for the prognostic value of RCC2 (online supplemental table 6). RCC2 was estimated to contribute 9.6% of the relative proportion of explained variation in 5-year RFS (figure 2D). Although clinicopathological factors contributed with the majority (71.3%) of explained variation (figure 2D), RCC2 had a stronger prognostic impact than any of the other molecular variables (≤8.1% of explained variation). The relatively low number of patients with known *TP53* mutation status (a subset of the Norwegian series 2 only, N=347, table 1) precluded inclusion in multivariable analysis. However, RCC2 was found to be significantly associated with patient outcome only in *TP53* mutated (HR 2.58, 95% CI 1.34 to 4.95, p=0.0031, adj. p=0.025), not in *TP53* wild-type tumours (HR 1.06, 95% CI 0.47 to 2.39, p=0.89, adj. p=1) (p_interaction_=0.089, online supplemental figure 14).

## Discussion

Optimisation of adjuvant chemotherapy in stage II and III CRC is an ongoing debate,^30^ in particular for the subgroup of patients with MSS cancers. The decision to treat in this setting is currently based on tumour stage, colon or rectal cancer, prognostic factors and patient tolerability to treatment. Our results suggest that patients with stage III MSS CRC and low expression of the RCC2 protein may have superior benefit from adjuvant chemotherapy than patients with high RCC2 expression. We have previously reported that RCC2 is an independent prognostic biomarker in primary CRC (in Norwegian series 1).^19^ In the present study, this was validated in an independent series from the same hospital, but only in patients who did not receive adjuvant chemotherapy. There was a statistically significant interaction between RCC2 and chemotherapy for outcome in the combined Norwegian series, implying that a low expression of RCC2 was predictive of the effect of chemotherapy in this retrospective analysis of an observational study cohort treated according to national guidelines. In support of this predictive effect, preclinical studies of cell line models from ovarian, lung, cervical and breast cancer have demonstrated an association between high expression of RCC2 and resistance to chemotherapy.^17 21^

We could only analyse interactions between RCC2 and chemotherapy in patients with stage III CRC, due to the low number of stage I and II patients who received such treatment. However, the association between low RCC2 expression and poor prognosis in chemotherapy-untreated cancers was strong also in stage II (online supplemental figure 15), suggesting a potential to improve the outcome of patients in the low-RCC2 subgroup also in this stage. In fact, RCC2 was found to be an independent prognostic biomarker in multivariable analysis of chemotherapy-untreated stage I–III patients, after adjusting for TNM stage, patient age, sex, tumour location, MSI status, *CDX2* expression, *BRAF*^V600E^- and *KRAS* mutation status. A limitation in this analysis was the missing information on the molecular variables *CDX2*, *BRAF*^V600E^ and *KRAS* for a proportion of the patients. However, results for RCC2 analysed in a multivariable model excluding these variables, which allowed for inclusion of substantially more patients, were virtually identical. Furthermore, the association between RCC2 and benefit from chemotherapy was independent of treatment regimen, and the results were similar for 5-fluorouracil monotherapy or in combination with oxaliplatin. Loss of CDX2 expression has been proposed as a biomarker for benefit from chemotherapy in CRC, also in our patient cohorts.^11 12^ RCC2 seemed to be independent of CDX2 in this setting, but due to the low number of CDX2 negative cases, we cannot firmly conclude on the prognostic relationship between RCC2, CDX2 and adjuvant chemotherapy.

A study by Song *et al* has reported that wild-type TP53 may transcriptionally activate RCC2, which in turn inhibits RAC1 and thus prevents formation of metastases.^20^ Although these results are interesting, we did not find any differences in RCC2 expression levels related to *TP53* mutational status. However, we did find low RCC2 expression to be associated with a poor patient prognosis only in *TP53* mutated, and not in *TP53* wild-type tumours. It is important to note that in our study we analysed cytoplasmic RCC2 expression, since this was found to be a stronger predictor of patient outcome than nuclear expression.^19^ It is possible that this relates to the interaction RCC2 has with RAC1 in the cytoplasm, in contrast to its role in the nucleus where RCC2 regulates the chromosomal passenger complex. However, the nuclear and cytoplasmic expressions of RCC2 were strongly correlated, and additional studies are needed to clarify the functional relationship between RCC2 and its biomarker value. Notably, high expression of RCC2 has been linked to epithelial-mesenchymal transition and worse outcome in other cancer types, including lung and breast cancer, suggesting that the effect of RCC2 might be cancer type-dependent.^16 31^

In the current study, we developed and employed a new methodological approach for in situ analysis of RCC2 by IHC, including the use of a validated monoclonal antibody, which facilitates potential future use in the clinic. We used fluorescent staining and digital image analysis, which together offer continuous scoring of biomarkers, and increases throughput of TMA-analyses by eliminating the process of visual scoring. Considering the different scale of these continuous data compared with the categorised RCC2 expression data provided by DAB-staining and visual analysis by the Allred method, we did not perform benchmarking of the two methods. However, due to the large dynamic linear range and continuous scoring scale provided by the new method, this approach is well suited for accurate and objective biomarker analyses.^32 33^

In summary, we have shown that low expression of RCC2 is a biomarker for a poor prognosis in non-metastatic CRC, and our data suggest that it may also be a predictive marker for a survival benefit from adjuvant chemotherapy.
