# Supplementary material for: Prediction of relapse-free survival according to adjuvant chemotherapy and regulator of chromosome condensation 2 (RCC2) expression in colorectal cancer
Source: ESMO Open. 2020 Nov 20;5(6):e001040. doi: 10.1136/esmoopen-2020-001040 (PMC7682466; doi:10.1136/esmoopen-2020-001040)
Supplement: Supplementary data [file esmoopen-2020-001040supp001.pdf]

Supplementary Material

Bergsland CH, *et al. ESMO Open* 2020; 5:e001040. doi: 10.1136/esmoopen-2020-001040

Table of contents

Supplementary Material 1

Supplementary figure 1: Flow-diagram of patient-samples from the three tissue-microarrays that were included/excluded for the various analyses. 4

Supplementary figure 2: Overview of methodology used for analysis of the three cohorts. 5

Supplementary figure 3: Five-year RFS of R0-resected stage I-III CRC patients of the two Norwegian series. 6

Supplementary figure 4: Associations between RCC2 and five-year RFS according to cellular localization. 7

Supplementary figure 5: Five-year RFS according to RCC2 and stage in the QUASAR2 cohort. 8

Supplementary figure 6: Five-year RFS according to RCC2 and treatment regimen in QUASAR2.. 9

Supplementary figure 7: Five-year RFS according to RCC2 and type of chemotherapy treatment in stage I-III patients of the pooled Norwegian series. 10

Supplementary figure 8: Five-year RFS according to RCC2 status in stage I-III patients who did not receive adjuvant chemotherapy, split by series. 11

Supplementary figure 9: Five-year RFS in stage III patients with and without adjuvant chemotherapy and according to RCC2 expression in Norwegian series 1 (A) and Norwegian series 2 (B). 12

Supplementary figure 10: Five-year RFS in stage III MSI patients with and without adjuvant chemotherapy and according to RCC2 expression in the pooled Norwegian series. 13

Supplementary figure 11: Five-year RFS according to RCC2 status in stage I-III colon cancer patients of the pooled Norwegian series (A), the QUASAR2 cohort (B), and in the pooled Norwegian series split by adjuvant chemotherapy (C). 14

Supplementary figure 12: Benefit of chemotherapy according to RCC2 status in stage III colon cancer patients of the pooled Norwegian series. 15

Supplementary figure 13: Five-year RFS in stage III patients with and without adjuvant chemotherapy, according to RCC2 and CDX2 expression in the pooled Norwegian series. 16

Supplementary figure 14: Association between RCC2 and five-year RFS according to TP53 mutation status. 17

Supplementary figure 15: Five-year RFS according to RCC2 expression and TNM stage in chemotherapy untreated patients of the pooled Norwegian series 18

Supplementary table 1: REMARK checklist. 19

Supplementary table 2: Adjuvant chemo- and radiotherapy characteristics in R0-resected stage I-III CRC patients of the three cohorts. 20

Supplementary table 3: Associations between RCC2 and molecular variables according to the two different methods used to analyze the Norwegian series 1. 21

Supplementary table 4: Clinicopathological data for all patients in the two Norwegian series and associations to RCC2 scores. 22

Supplementary table 5: Clinicopathological data for all patients in the QUASAR2 series and their relation to categorized RCC2 scores. 24

Supplementary table 6: Multivariable five-year relapse-free survival analysis in stage I-III chemotherapy untreated patients in the pooled Norwegian series, excluding *KRAS*, *BRAF*^{V600E} and CDX2 as covariables. 25

Supplementary table 7: Complementary analyses with RCC2 modeled as a continuous linear variable. 26

Supplementary Methods 27

Supplementary References 29

Supplementary figure 1: Flow-diagram of patient-samples from the three tissue-microarrays that were included/excluded for the various analyses.

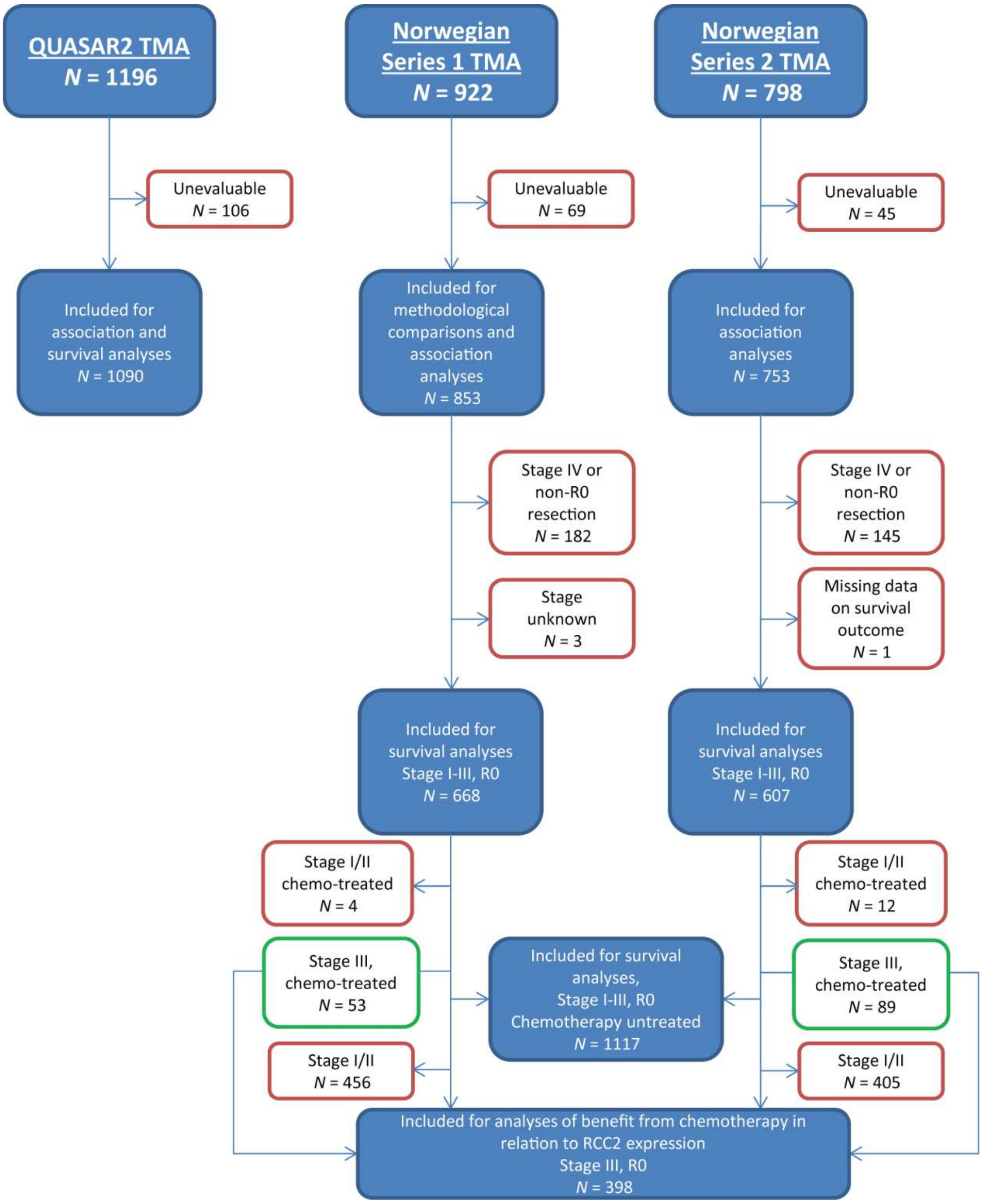

Bergsland CH, *et al. ESMO Open* 2020; 5:e001040. doi: 10.1136/esmoopen-2020-001040

Supplementary figure 2: Overview of methodology used for analysis of the three cohorts. Abbreviations: DAB; 3,3-diaminobenzidine, RCC2; Regulator of chromosome condensation 2, pctl; percentile. *Data previously published in [1].

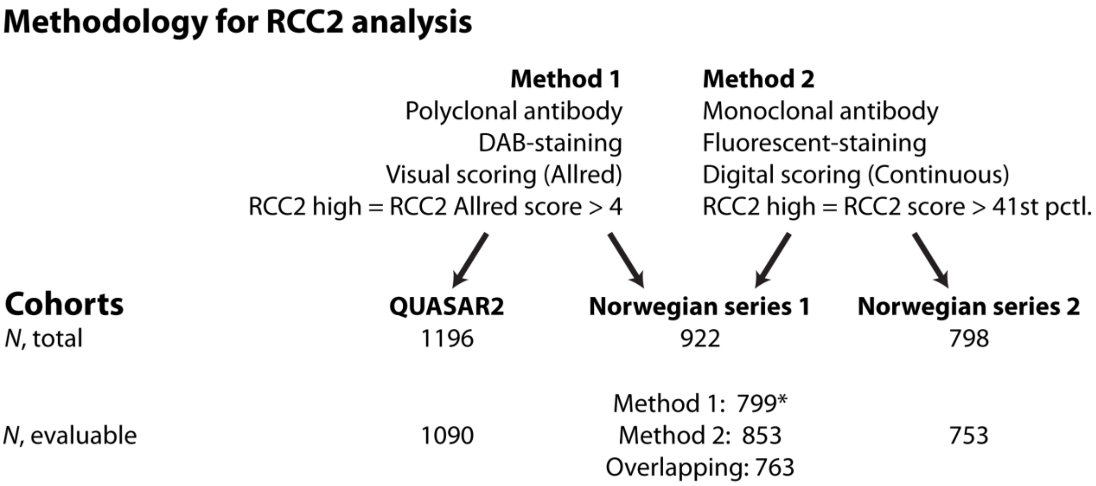

Supplementary figure 3: Five-year RFS of R0-resected stage I-III CRC patients of the two Norwegian series.

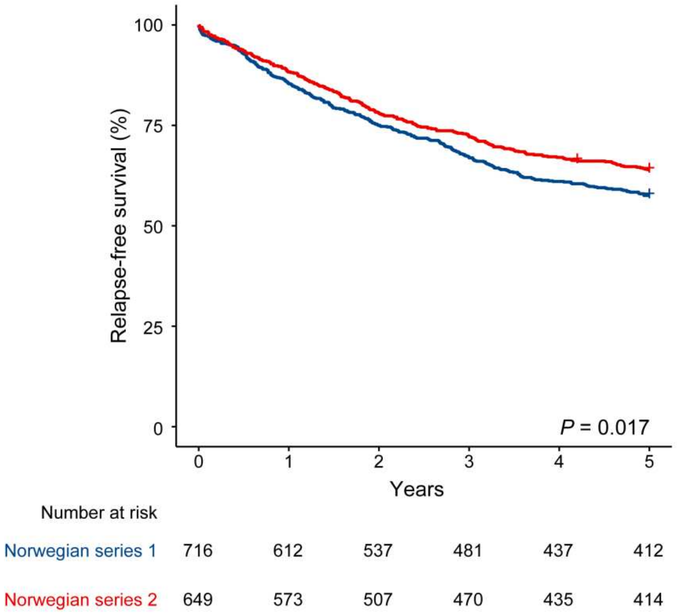

Supplementary figure 4: Associations between RCC2 and five-year RFS according to cellular localization. A) The continuous nuclear and cytoplasmic RCC2 scores are correlated. B) Both nuclear and cytoplasmic expression of RCC2 were dichotomized at the 41st percentile of scores in the Norwegian series 1, and analyzed by Kaplan-Meier survival analysis. Although both measures showed an association between low expression of RCC2 and poor patient outcome, cytoplasmic expression was a stronger predictor.

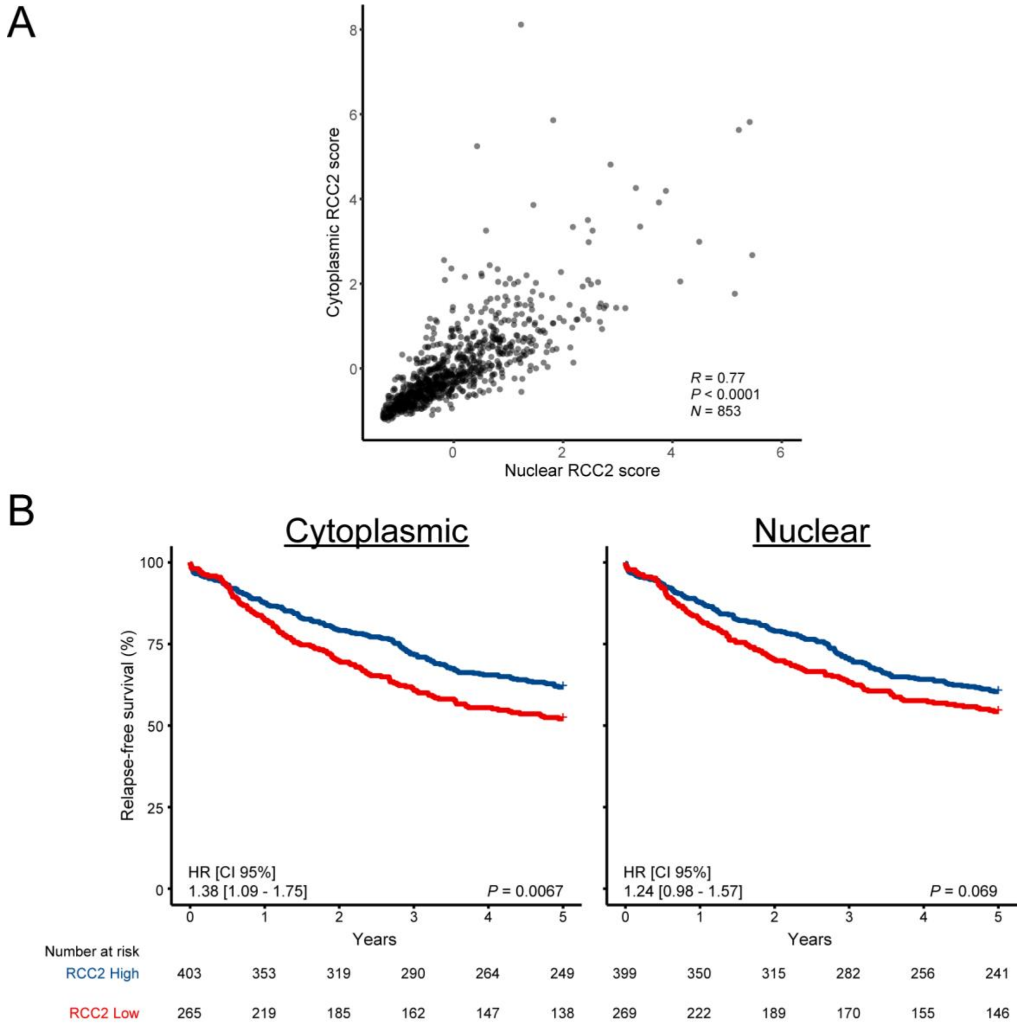

Bergsland CH, *et al. ESMO Open* 2020; 5:e001040. doi: 10.1136/esmoopen-2020-001040

Supplementary figure 5: Five-year RFS according to RCC2 and stage in the QUASAR2 cohort.

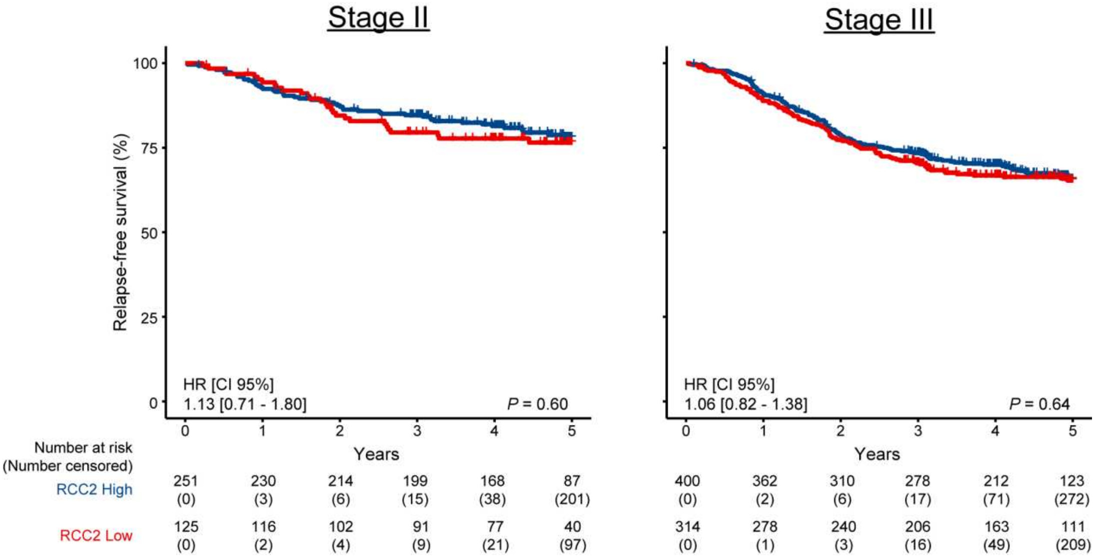

Bergsland CH, *et al. ESMO Open* 2020; 5:e001040. doi: 10.1136/esmoopen-2020-001040

Supplementary figure 6: Five-year RFS according to RCC2 and treatment regimen in QUASAR2.

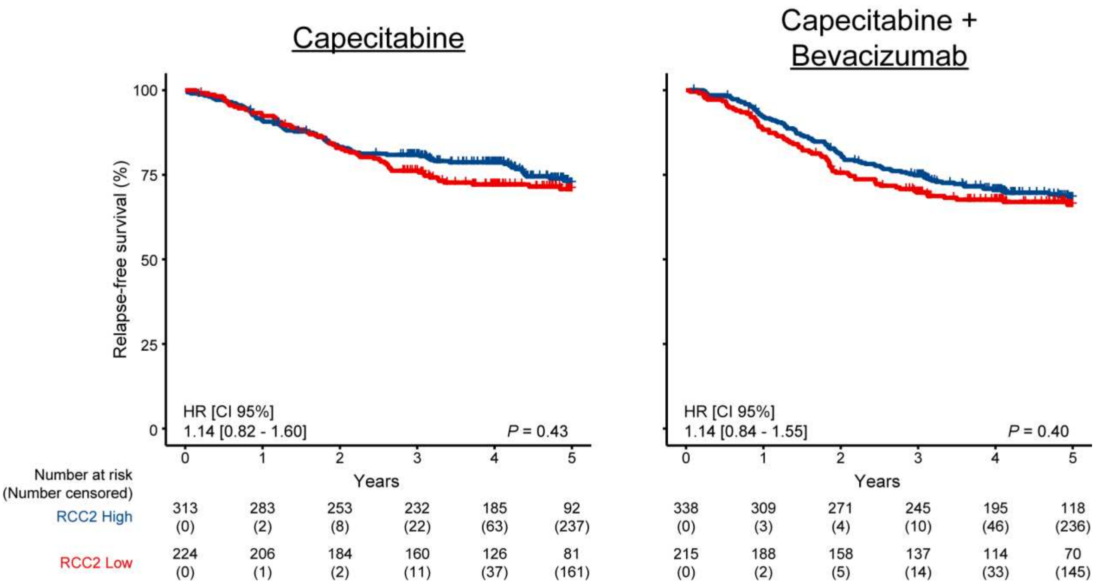

Supplementary figure 7: Five-year RFS according to RCC2 and type of chemotherapy treatment in stage I-III patients of the pooled Norwegian series. Patients were split into groups receiving FLOX (fluorouracil, leucovorin and oxaliplatin, left panel), FLV (fluorouracil and leucovorin, middle panel) and unknown or other treatment regimen (right panel).

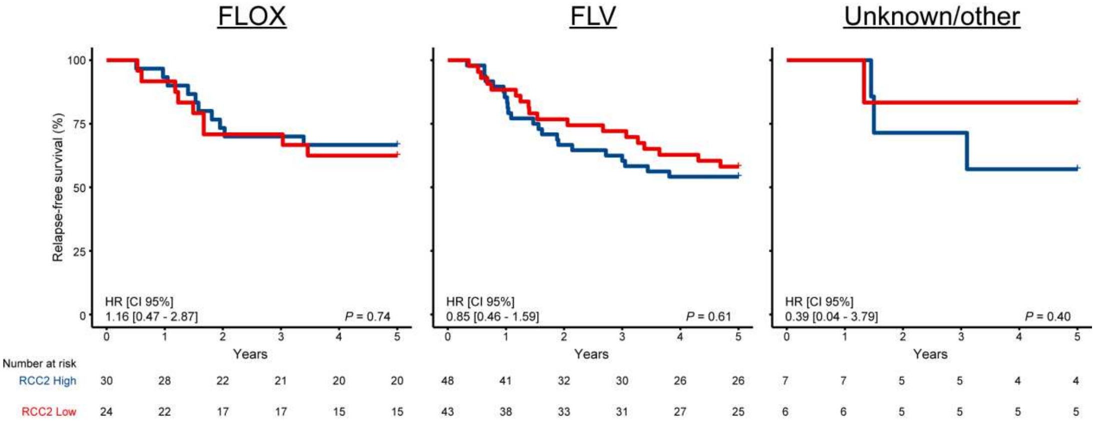

Supplementary figure 8: Five-year RFS according to RCC2 status in stage I-III patients who did not receive adjuvant chemotherapy, split by series.

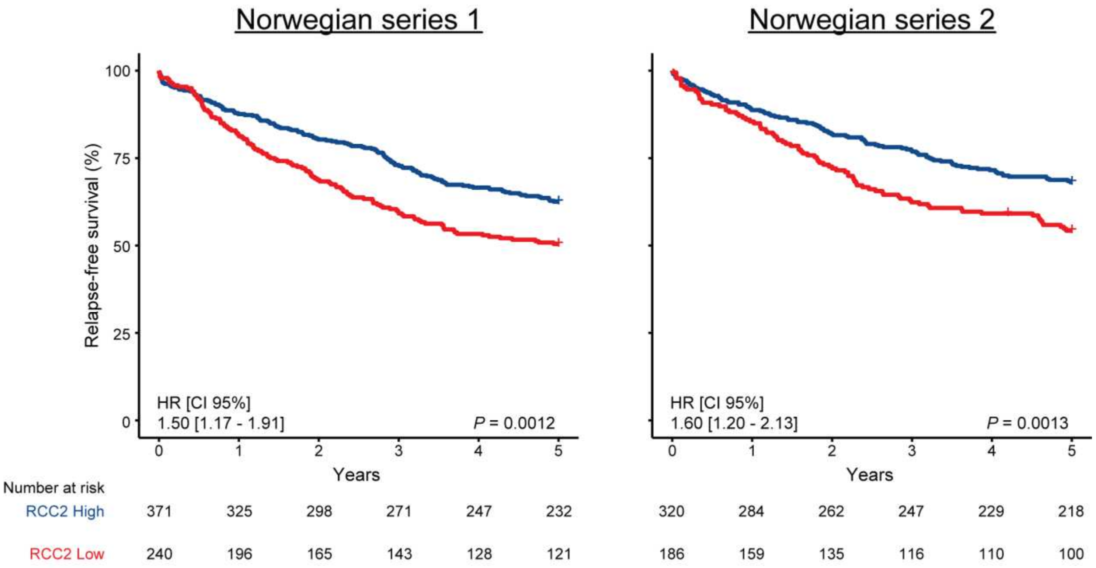

Bergsland CH, *et al. ESMO Open* 2020; 5:e001040. doi: 10.1136/esmoopen-2020-001040

Supplementary figure 9: Five-year RFS in stage III patients with and without adjuvant chemotherapy and according to RCC2 expression in Norwegian series 1 (A) and Norwegian series 2 (B).

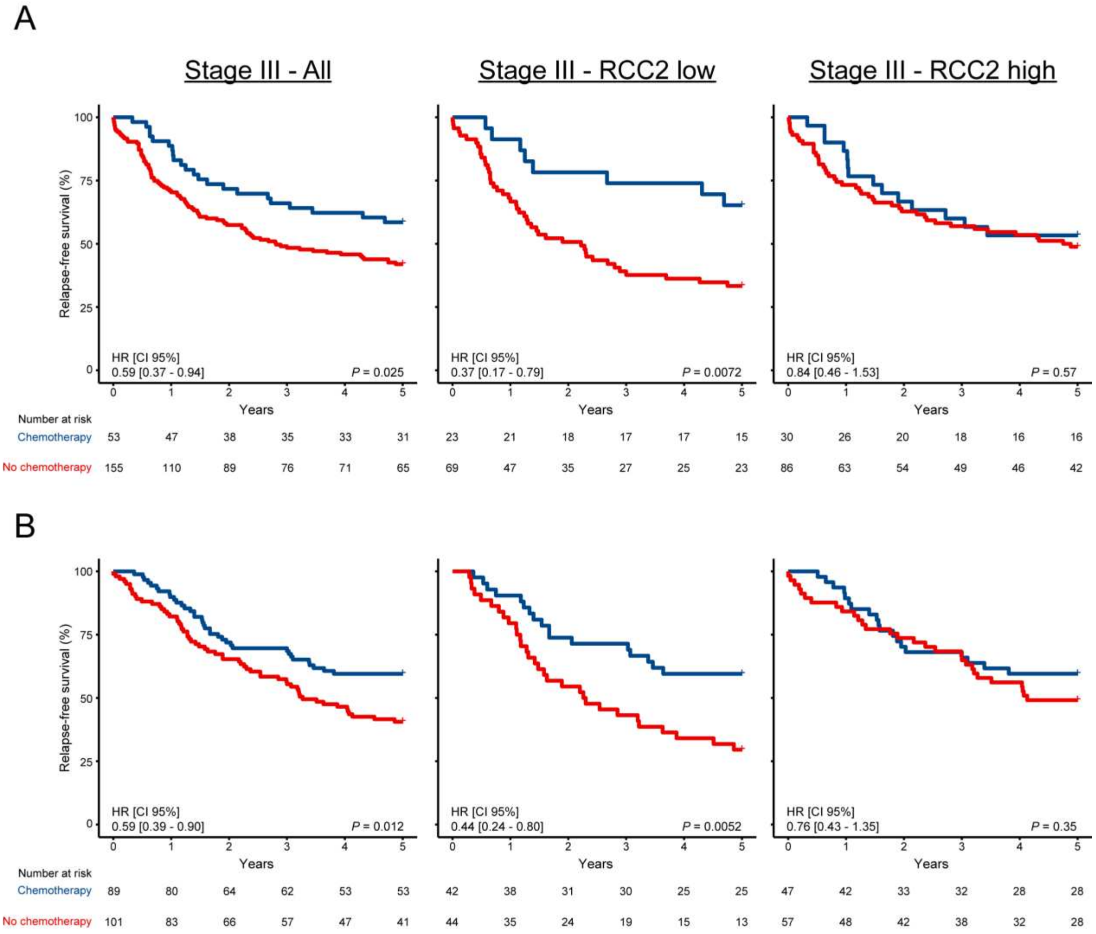

Bergsland CH, *et al. ESMO Open* 2020; 5:e001040. doi: 10.1136/esmoopen-2020-001040

Supplementary figure 10: Five-year RFS in stage III MSI patients with and without adjuvant chemotherapy and according to RCC2 expression in the pooled Norwegian series.

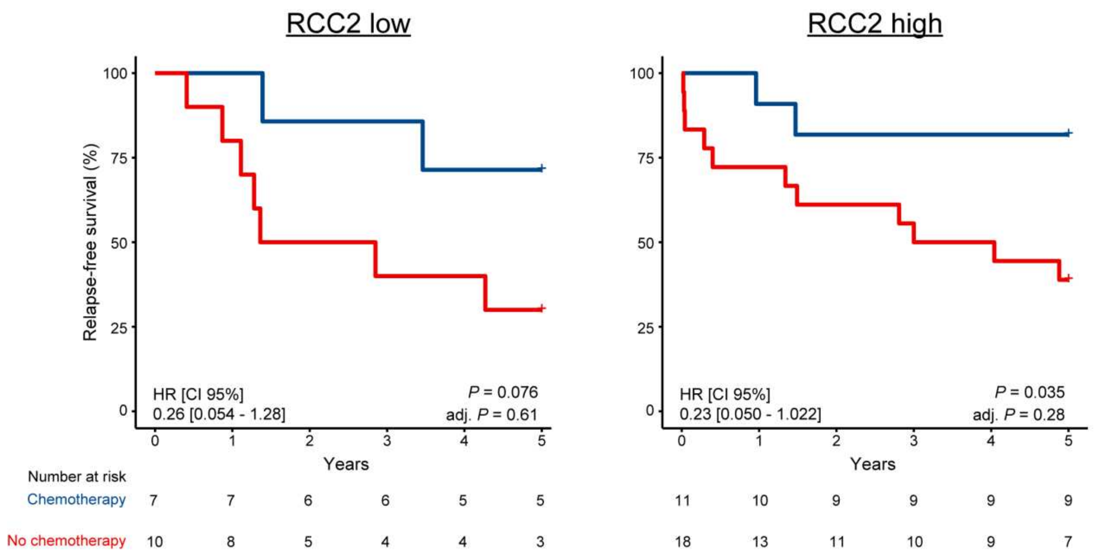

Supplementary figure 11: Five-year RFS according to RCC2 status in stage I-III colon cancer patients of the pooled Norwegian series (A), the QUASAR2 cohort (B), and in the pooled Norwegian series split by adjuvant chemotherapy (C).

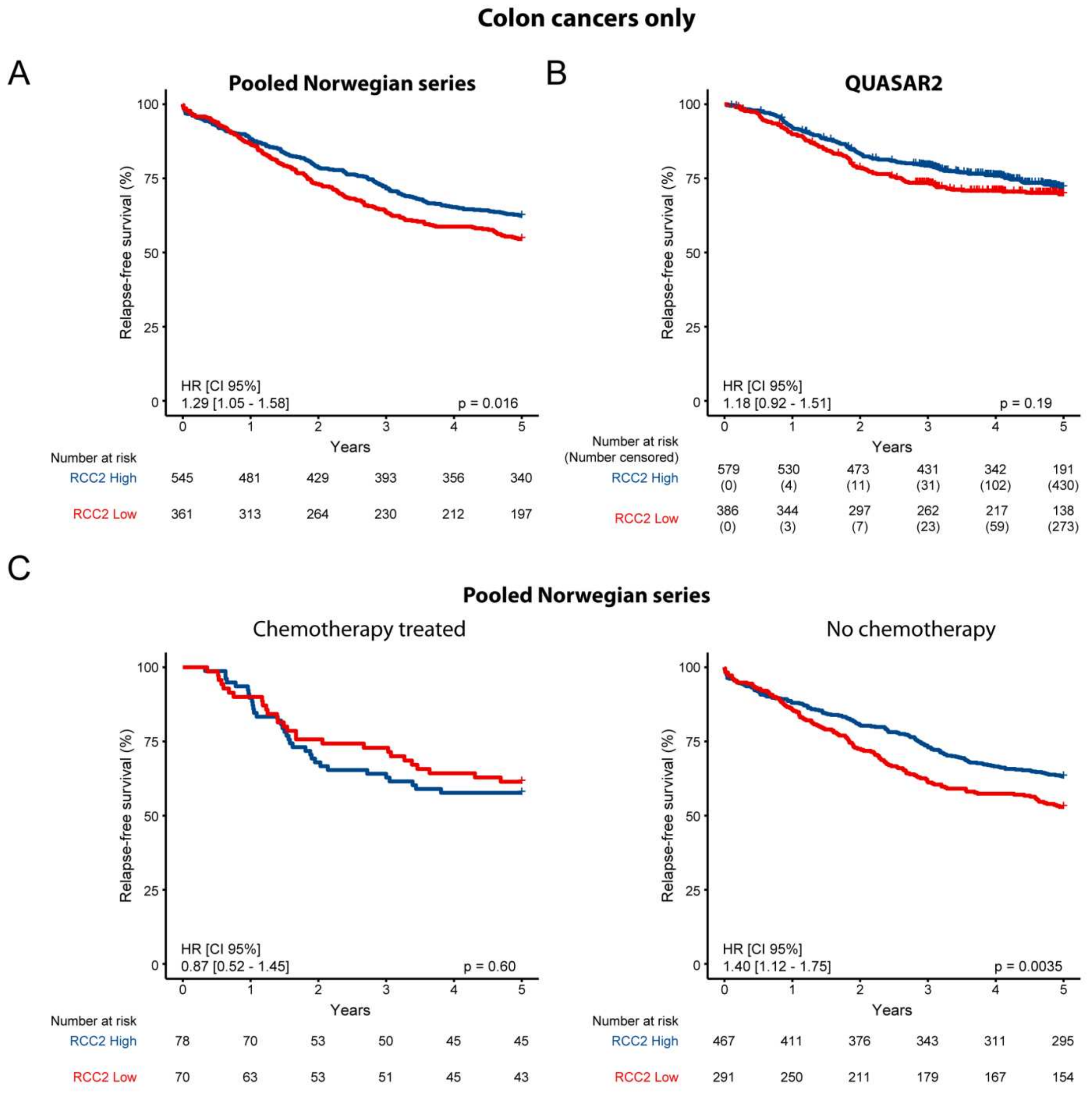

Supplementary figure 12: Benefit of chemotherapy according to RCC2 status in stage III colon cancer patients of the pooled Norwegian series. Benefit of adjuvant chemotherapy was assessed in all stage III colon cancer patients (A), according to low/high RCC2 (B) and according to low/high RCC2 in MSS patients only. Abbreviations: CC; Colon cancer, MSS; microsatellite stable.

A

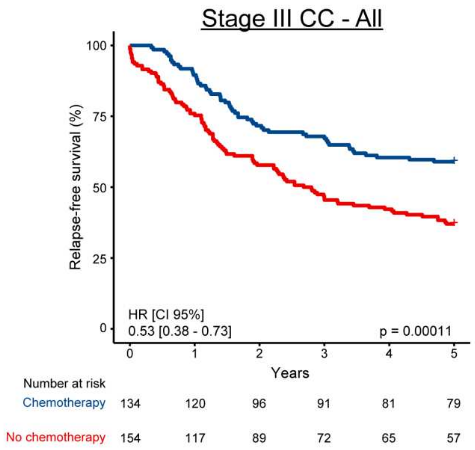

B

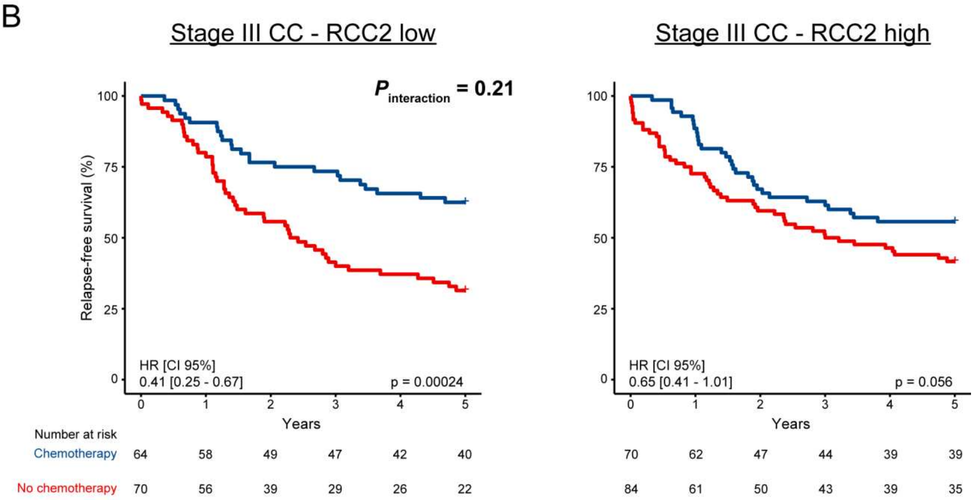

C

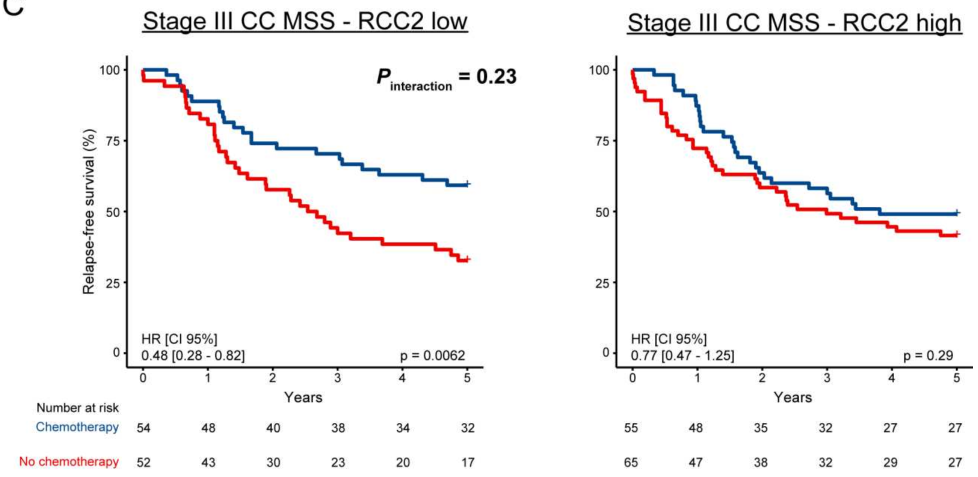

Supplementary figure 13: Five-year RFS in stage III patients with and without adjuvant chemotherapy, according to RCC2 and CDX2 expression in the pooled Norwegian series.

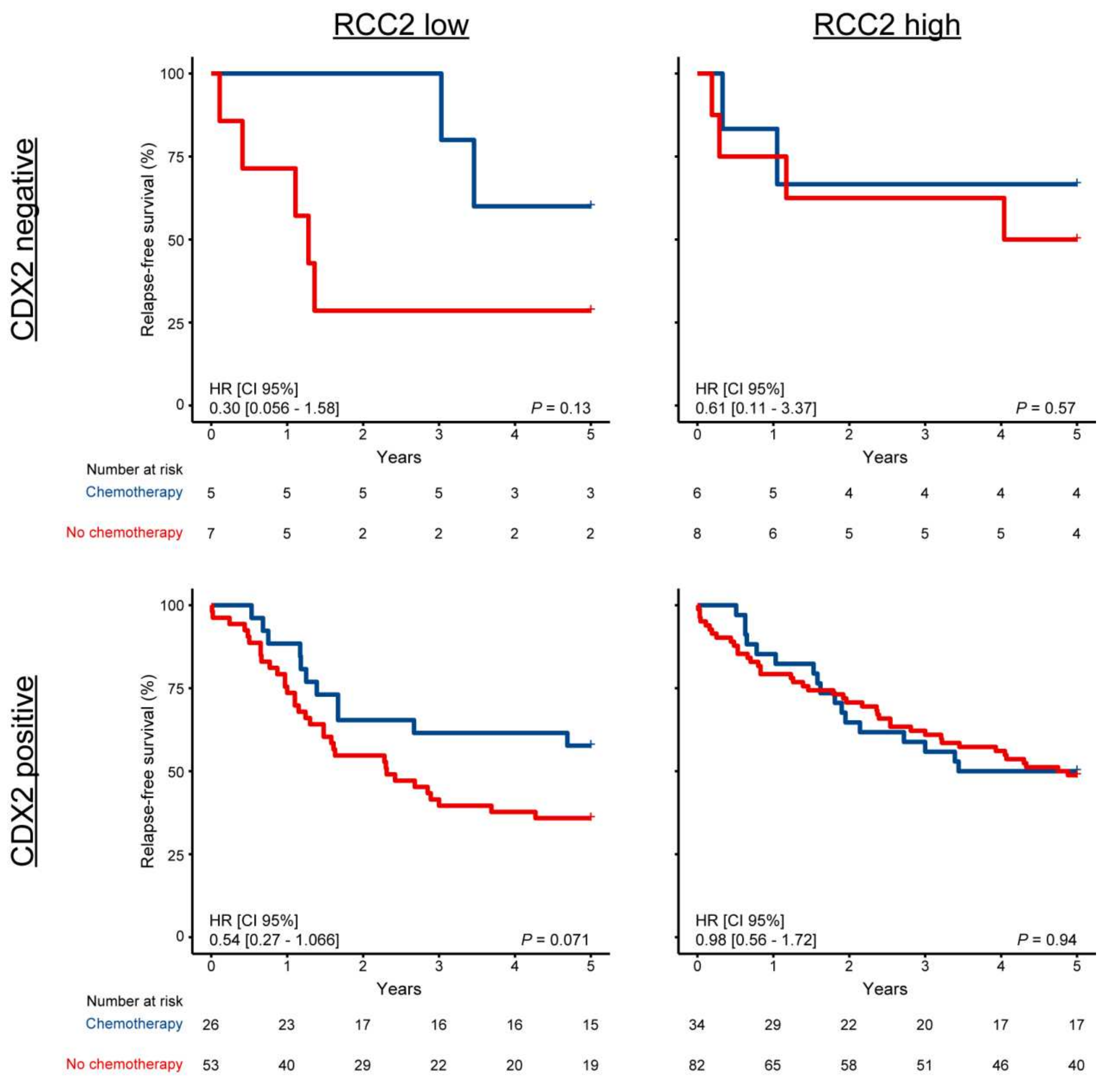

Supplementary figure 14: Association between RCC2 and five-year RFS according to TP53 mutation status. Results are from analyses of patients in the Norwegian series 2 with stage I-III CRC who did not receive adjuvant chemotherapy, and who had available *TP53* mutational data (N = 228).

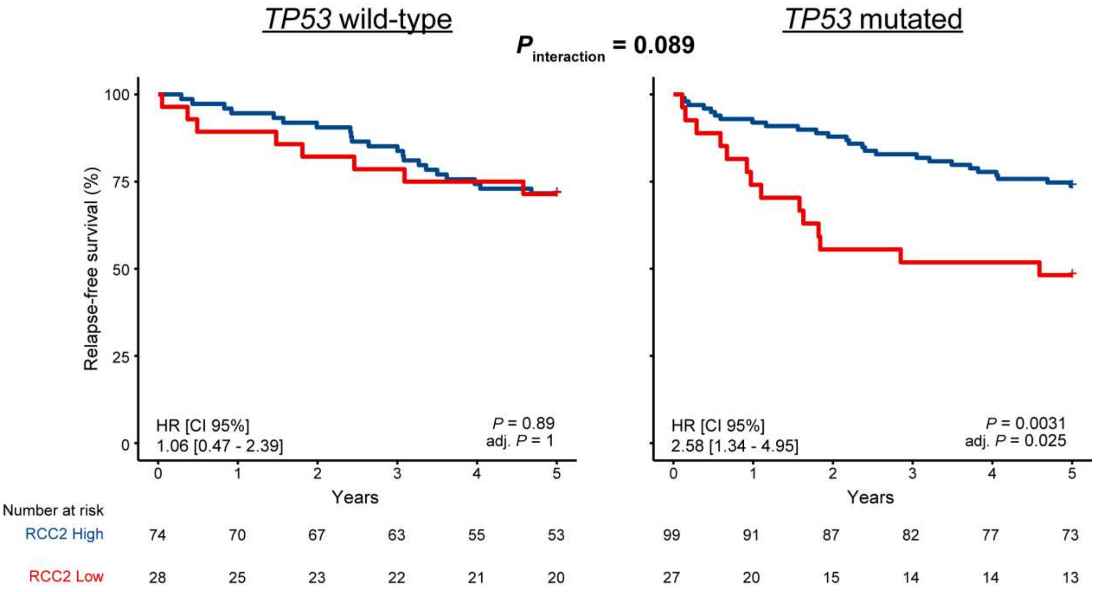

Bergsland CH, *et al. ESMO Open* 2020; 5:e001040. doi: 10.1136/esmoopen-2020-001040

Supplementary figure 15: Five-year RFS according to RCC2 expression and TNM stage in chemotherapy untreated patients of the pooled Norwegian series (A). Analysis was further stratified by pT (B) and tumor differentiation (C) in stage II patients. Information on pT was missing for three patients, and tumor differentiation was missing for 16 patients.

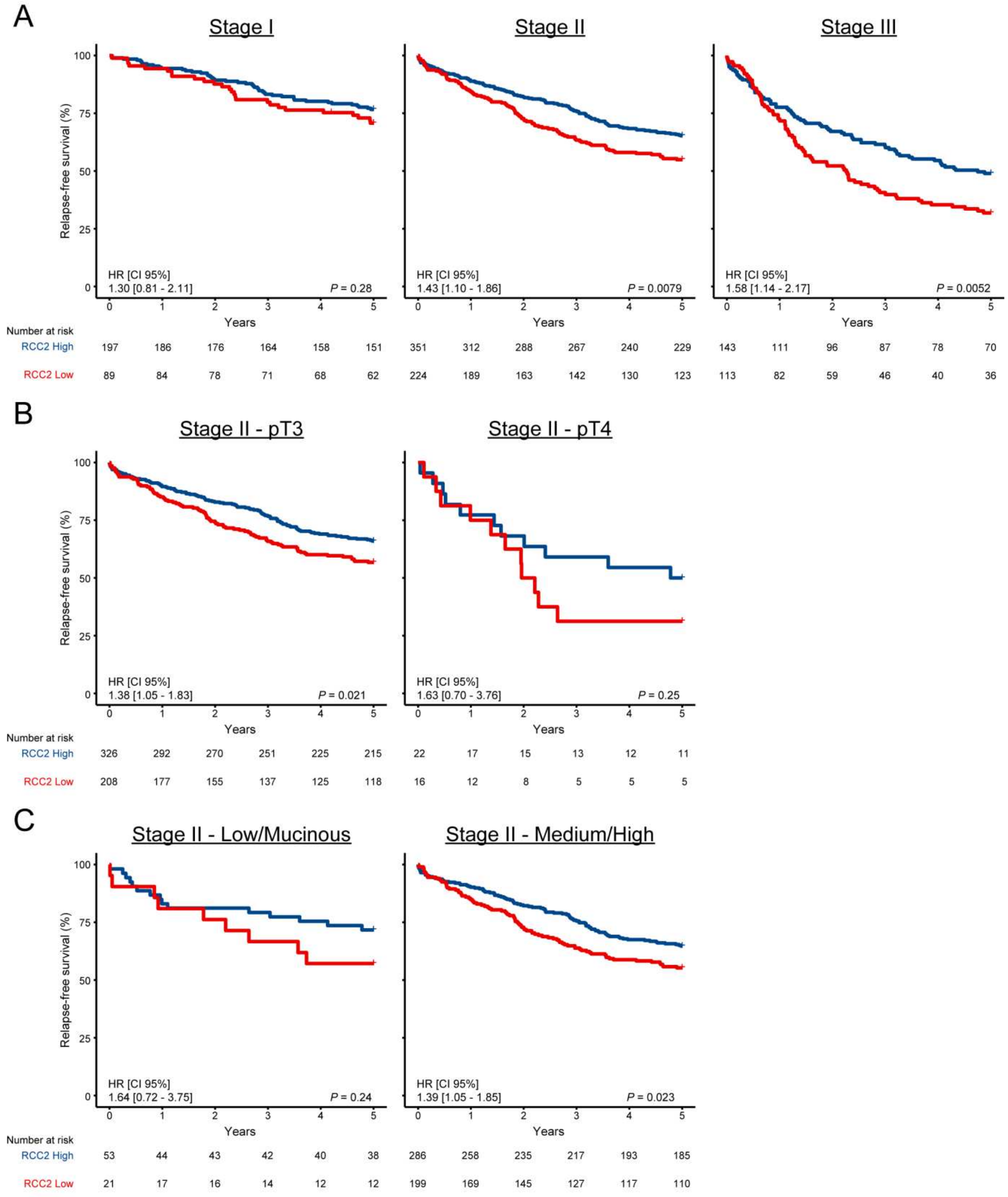

Supplementary table 1: REMARK checklist. [2]

	Item to be reported	Page no.
**INTRODUCTION**		
1	State the marker examined, the study objectives, and any pre-specified hypotheses.	2
**MATERIALS AND METHODS**		
Patients		
2	Describe the characteristics (e.g., disease stage or co-morbidities) of the study patients, including their source and inclusion and exclusion criteria.	2-4 & Table 1
3	Describe treatments received and how chosen (e.g., randomized or rule-based).	2
Specimen characteristics		
4	Describe type of biological material used (including control samples) and methods of preservation and storage.	2-4
Assay methods		
5	Specify the assay method used and provide (or reference) a detailed protocol, including specific reagents or kits used, quality control procedures, reproducibility assessments, quantitation methods, and scoring and reporting protocols. Specify whether and how assays were performed blinded to the study endpoint.	4-5 & Supp. methods
Study design		
6	State the method of case selection, including whether prospective or retrospective and whether stratification or matching (e.g., by stage of disease or age) was used. Specify the time period from which cases were taken, the end of the follow-up period, and the median follow-up time.	2
7	Precisely define all clinical endpoints examined.	4
8	List all candidate variables initially examined or considered for inclusion in models.	4-5 & Table 1
9	Give rationale for sample size; if the study was designed to detect a specified effect size, give the target power and effect size.	2
Statistical analysis methods		
10	Specify all statistical methods, including details of any variable selection procedures and other model-building issues, how model assumptions were verified, and how missing data were handled.	4-5
11	Clarify how marker values were handled in the analyses; if relevant, describe methods used for cutpoint determination.	4-5
**RESULTS**		
Data		
12	Describe the flow of patients through the study, including the number of patients included in each stage of the analysis (a diagram may be helpful) and reasons for dropout. Specifically, both overall and for each subgroup extensively examined report the numbers of patients and the number of events.	S.Fig. 1
13	Report distributions of basic demographic characteristics (at least age and sex), standard (disease-specific) prognostic variables, and tumor marker, including numbers of missing values.	Table 1
Analysis and presentation		
14	Show the relation of the marker to standard prognostic variables.	Tables S4,S5
15	Present univariable analyses showing the relation between the marker and outcome, with the estimated effect (e.g., hazard ratio and survival probability). Preferably provide similar analyses for all other variables being analyzed. For the effect of a tumor marker on a time-to-event outcome, a Kaplan-Meier plot is recommended.	Table 2 & figures 1,2,3
16	For key multivariable analyses, report estimated effects (e.g., hazard ratio) with confidence intervals for the marker and, at least for the final model, all other variables in the model.	Table 2
17	Among reported results, provide estimated effects with confidence intervals from an analysis in which the marker and standard prognostic variables are included, regardless of their statistical significance.	Table 2
18	If done, report results of further investigations, such as checking assumptions, sensitivity analyses, and internal validation.	5
**DISCUSSION**		
19	Interpret the results in the context of the pre-specified hypotheses and other relevant studies; include a discussion of limitations of the study.	10
20	Discuss implications for future research and clinical value.	10

Bergsland CH, *et al. ESMO Open* 2020; 5:e001040. doi: 10.1136/esmoopen-2020-001040

Supplementary table 2: Adjuvant chemo- and radiotherapy characteristics in R0-resected stage I-III CRC patients of the three cohorts.

		Norwegian series 1 (1993-2003)	Norwegian series 2 (2003-2012)	QUASAR2 (2005-2010)
	**R0-resected stage I-III CRC patients**			
**Adjuvant chemotherapy (yes/total), according to TNM stage**	I	0/136 (0%)	1/167 (0.6%)	-
	II	4/357 (1%)	12/281 (4%)	420/420 (100%)
	III	58/223 (26%)	93/202 (46%)	776/776 (100%)
	Total	62/716 (9%)	106/650 (16%)	1196 (100%)
**Type of chemotherapy**	Nordic FLV (5-fluorouracil and leucovorin)	58 (94%)	38 (36%)	-
	FLOX (5-fluorouracil, leucovorin, oxaliplatin)	1 (2%)	58 (55%)	-
	Capecitabine	-	-	587 (49%)
	Capecitabine and bevacizumab	-	-	609 (51%)
	Unknown/Other	3 (5%)	10 (9%)	-
	**R0-resected stage I-III rectal cancer patients**			
**Pre-operative radiotherapy**	No	181 (92%)	136 (76%)	-*
	Yes	15 (8%)	44 (24%)	-*
**Post-operative radiotherapy**	No	149 (96%)	161 (98%)	-*
	Yes	6 (4%)	3 (2%)	-*
	NA	41	16	-*

*Neoadjuvant radiotherapy or expected need for radiotherapy within 12 months of treatment were exclusion criteria in the QUASAR2 trial.

Bergsland CH, *et al. ESMO Open* 2020; 5:e001040. doi: 10.1136/esmoopen-2020-001040

Supplementary table 3: Associations between RCC2 and molecular variables according to the two different methods used to analyze the Norwegian series 1. Only overlapping samples were included and dichotomized RCC2 scores were used in the analysis. The Fisher Exact test was used to test for associations between RCC2 and each variable. NAs were not included in table analysis. Abbreviations: MSI; microsatellite instable, MSS; microsatellite stable, NA; not available.

		**Allred scoring, polyclonal antibody**			**Digital scoring, monoclonal antibody**		
	**Total**	**RCC2 Low**	**RCC2 High**	***P***	**RCC2 Low**	**RCC2 High**	***P***
**Patients**	763	312 (41%)	451 (59%)		304 (40%)	459 (60%)	
**MSI status**				**0.0044**			**0.060**
MSI	102 (15%)	28 (27%)	74 (73%)		30 (29%)	72 (71%)	
MSS	594 (85%)	254 (43%)	340 (57%)		235 (40%)	359 (60%)	
NA	67	30	37		39	28	
***BRAF* mutational status**				**0.15**			**0.26**
Wild-type	538 (84%)	215 (40%)	323 (60%)		206 (38%)	332 (62%)	
Mutated	100 (16%)	32 (32%)	68 (68%)		32 (32%)	68 (68%)	
NA	125	65	60		66	59	
***KRAS* mutational status**				**0.13**			**0.51**
Wild-type	379 (68%)	133 (35%)	246 (65%)		131 (35%)	248 (65%)	
Mutated	176 (32%)	74 (42%)	102 (58%)		66 (37.5%)	110 (62.5%)	
NA	208	105	103		107	101	
***CDX2* expression**				**0.41**			**1**
Positive	513 (90%)	223 (43%)	290 (57%)		207 (40%)	306 (60%)	
Negative	59 (10%)	22 (37%)	37 (63%)		24 (41%)	35 (59%)	
NA	191	67	124		73	118	

Bergsland CH, *et al. ESMO Open* 2020; 5:e001040. doi: 10.1136/esmoopen-2020-001040

Supplementary table 4: Clinicopathological data for all patients in the two Norwegian series and associations to RCC2 scores. Patient characteristics are stratified according to dichotomized RCC2 scores. *P*-values for tests of associations between RCC2 and each patient characteristic are based on the continuous RCC2-scores. Pearson's correlation was used to test for an association between RCC2 and patient age. The Wilcoxon test was used to test for associations between RCC2 and patient sex, MSI-status, *BRAF/KRAS/TP53* mutational status, CDX2 expression status and treatment with chemotherapy. The Kruskal-Wallis test was used to test for associations between RCC2 and tumor stage and location. NAs not included in table analysis. Abbreviations: MSI; microsatellite instable, MSS; microsatellite stable, NA; not available.

	**Norwegian Series 1**					**Norwegian Series 2**				
	**Total**	**RCC2 Low**	**RCC2 High**	NA	***P***	**Total**	**RCC2 Low**	**RCC2 High**	NA	***P***
**Patients**	922	353 (41%)	500 (59%)	69		798	309 (41%)	444 (59%)	45	
**Age**					**0.13**					**0.91**
Median (range)	73 (29–94)	73 (29–91)	73 (30–94)	73 (34–89)		72 (27–97)	72 (27–97)	73 (27–93)	65 (41–93)	
**Sex**					**0.79**					**0.69**
Female	485 (53%)	183 (41%)	266 (59%)	36		407 (51%)	160 (41%)	232 (59%)	15	
Male	437 (47%)	170 (42%)	234 (58%)	33		391 (49%)	149 (41%)	212 (59%)	30	
**TNM stage**					**0.0001**					**<0.0001**
I	137 (15%)	41 (31%)	90 (69%)	6		167 (21%)	49 (31%)	108 (69%)	10	
II	381 (41%)	145 (42%)	204 (58%)	32		288 (36%)	101 (38%)	166 (62%)	21	
III	242 (26%)	98 (44%)	126 (56%)	18		214 (27%)	93 (46%)	110 (54%)	11	
IV	159 (17%)	69 (47%)	77 (53%)	13		129 (16%)	66 (52%)	60 (48%)	3	
NA	3	0	3	0		-	-	-	-	
**Tumor location**					**0.55**					**0.87**
Right colon	365 (40%)	142 (42%)	196 (58%)	27		327 (41%)	130 (41%)	186 (59%)	11	
Left colon	301 (32%)	115 (41%)	164 (59%)	22		239 (30%)	108 (47%)	121 (53%)	10	
Rectum	231 (25%)	86 (40%)	127 (60%)	18		218 (27%)	66 (34%)	128 (66%)	24	
Synchronous	25 (3%)	10 (43%)	13 (57%)	2		14 (2%)	5 (36%)	9 (64%)	0	
**MSI status**					**0.27**					**0.0042**
MSI	128 (15%)	37 (32%)	77 (68%)	14		120 (16%)	35 (30%)	81 (70%)	4	
MSS	712 (85%)	273 (41%)	393 (59%)	46		638 (84%)	265 (43%)	357 (57%)	16	
NA	82	43	30	9		40	9	6	25	
***BRAF*^{V600E} mutational status**					**0.69**					**0.90**
Wild-type	650 (85%)	241 (40%)	360 (60%)	49		288 (83%)	76 (28%)	196 (72%)	16	
Mutated	119 (15%)	38 (34%)	74 (66%)	7		57 (17%)	17 (31%)	38 (69%)	2	
NA	153	74	66	13		453	216	210	27	
***KRAS* mutational status**					**0.50**					**0.59**

Bergsland CH, *et al. ESMO Open* 2020; 5:e001040. doi: 10.1136/esmoopen-2020-001040

Wild-type	463 (69%)	154 (36%)	269 (64%)	40		238 (69%)	67 (29%)	162 (71%)	9	
Mutated	204 (31%)	74 (38%)	120 (62%)	10		106 (31%)	26 (27%)	72 (73%)	8	
NA	255	125	111	19		454	216	210	28	
***CDX2* expression**					**0.83**					**0.50**
Positive	568 (89%)	225 (41%)	323 (59%)	20		281 (89%)	77 (29%)	191 (71%)	13	
Negative	71 (11%)	26 (41%)	37 (59%)	8		34 (11%)	8 (24%)	26 (76%)	0	
NA	283	102	140	41		483	224	227	32	
***TP53* mutational status**				-						**0.58**
Wild-type	-	-	-			145 (42%)	41 (31%)	93 (69%)	11	
Mutated	-	-	-			202 (58%)	53 (27%)	142 (73%)	7	
NA	-	-	-			451	215	209	27	
**Chemotherapy Stage III, R0 patients**					**0.91**					**0.66**
No	165 (74%)	69 (45%)	86 (55%)	10		109 (54%)	45 (44%)	57 (56%)	7	
Yes	58 (26%)	23 (43%)	30 (57%)	5		93 (46%)	42 (47%)	47 (53%)	4	

Bergsland CH, *et al. ESMO Open* 2020; 5:e001040. doi: 10.1136/esmoopen-2020-001040

Supplementary table 5: Clinicopathological data for all patients in the QUASAR2 series and their relation to categorized RCC2 scores. *P*-values correspond to tests for associations between RCC2 expression and the respective patient characteristic. The Wilcoxon test was used to test for association between RCC2 and age. The chi-squared test was used to test for associations between RCC2 and tumor stage and location. The Fisher Exact test was used to test for associations between RCC2 and sex, MSI status, *BRAF*-mutational status, as well as treatment regimen. NAs were not included in table analysis. Abbreviations: MSI; microsatellite instable, MSS; microsatellite stable, NA; not available.

QUASAR2					
	**Total**	**RCC2 Low**	**RCC2 High**	NA	***P***
**Patients**	1196	439 (40%)	651 (60%)	106	
**Age**					**0.13**
Median (range)	65 (21–85)	65 (35–83)	64 (27–85)	66 (21–81)	
**Sex**					**0.12**
Female	507 (42%)	197 (43%)	261 (57%)	49	
Male	689 (58%)	242 (38%)	390 (62%)	57	
**TNM stage**					**0.00075**
II	420 (35%)	125 (33%)	251 (67%)	44	
III	776 (65%)	314 (44%)	400 (56%)	62	
**Tumor location**					**0.58**
Right colon	445 (42%)	173 (43%)	233 (57%)	39	
Left colon	490 (46%)	176 (39%)	272 (61%)	42	
Rectum	132 (12%)	53 (42%)	72 (58%)	7	
NA	129	37	74	18	
**MSI status**					**0.22**
MSI	154 (13%)	47 (35%)	87 (65%)	20	
MSS	988 (87%)	372 (41%)	537 (59%)	79	
NA	54	20	27	7	
***BRAF*^{V600E} mutational status**					**0.85**
Wild-type	956 (87%)	344 (40%)	525 (60%)	87	
Mutated	142 (13%)	55 (41%)	80 (59%)	7	
NA	98	40	46	12	
**Treatment**					**0.35**
Capecitabine	587 (49%)	224 (42%)	313 (58%)	50	
Capecitabine + Bevacizumab	609 (51%)	215 (39%)	338 (61%)	56	

Bergsland CH, *et al. ESMO Open* 2020; 5:e001040. doi: 10.1136/esmoopen-2020-001040

Supplementary table 6: Multivariable five-year relapse-free survival analysis in stage I-III chemotherapy untreated patients in the pooled Norwegian series, excluding *KRAS*, *BRAF*^{V600E} and CDX2 as covariables. Analysis was stratified by patient series.

Variable	Strata	Multivariable analysis* *N* = 1054, Events = 418	
		HR (95 % CI)	*P*
**RCC2**	High	1	-
	Low	1.42 (1.17 – 1.72)	0.00043
**Sex**	Female	1	-
	Male	1.23 (1.00 – 1.50)	0.045
**Age**	Below median**	1	-
	Above or equal to median**	1.87 (1.50 – 2.32)	<0.0001
**TNM stage**	I	1	-
	II	1.71 (1.29 – 2.27)	0.00017
	III	2.92 (2.18 – 3.92)	<0.0001
**Tumor location**	Right colon	1	-
	Left colon	1.22 (0.95 – 1.56)	0.12
	Rectum	1.12 (0.86 – 1.46)	0.41
	Synchronous	1.14 (0.63 – 2.05)	0.67
**MSI status**	MSI	1	-
	MSS	1.21 (0.89 – 1.64)	0.22

Abbreviations: MSI; microsatellite instable, MSS; microsatellite stable.
*Proportional-hazards assumption was violated. The model was therefore also evaluated with TNM stage and age as stratifying variables and results were similar (data not shown).
**Above/below median age (73) in the pooled series.

Supplementary table 7: Complementary analyses with RCC2 modeled as a continuous linear variable. RCC2 was scaled within each series and modeled as a continuous linear variable using cox proportional hazards models. The hazard ratio is calculated per unit increase in RCC2.

Analysis	Corresponds to analysis in figure/table	Range RCC2 (median)	Hazard ratio (95% CI), per unit increase in scaled RCC2 score	*P*
Norwegian series 1 stages I-III, univariable Cox model, RCC2, Five-year RFS	1D (right)	-1.2 – 5.8 (-0.20)	0.78 (0.66 – 0.92)	0.0025
Norwegian series 2 stages I-III, univariable Cox model, RCC2, Five-year RFS	2A	-1.0 – 7.2 (-0.27)	0.86 (0.75 – 1.00)	0.051
Pooled Norwegian series stages I-III, chemotherapy-treated patients, univariable Cox, RCC2, Five-year RFS	2C left	-1.1 – 5.6 (-0.38)	1.11 (0.87 – 1.41)	0.42
Pooled Norwegian series stages I-III, chemotherapy-untreated patients, univariable Cox, RCC2, Five-year RFS	2C right (+ table 2, univariable analysis)	-1.2 – 8.1 (-0.21)	0.81 (0.73 – 0.91)	0.00026
Pooled Norwegian series stage III, interaction between RCC2 and chemotherapy, Five-year RFS	3B	-1.2 – 5.8 (-0.36)	1.40 (1.01 – 1.94)	0.044
Pooled Norwegian series stage III MSS, interaction between RCC2 and chemotherapy, Five-year RFS	3C	-1.1 – 5.8 (-0.36)	1.50 (1.06 – 2.12)	0.023
Pooled Norwegian series stages I-III, multivariable Cox model in chemotherapy-untreated patients, RCC2, Five-year RFS	Table 2, multivariable analysis, Figure 2D	-1.2 – 7.2 (-0.04)	0.85 (0.73 – 0.99)<br><br>Relative proportion explained variation for RCC2 = 6.3%	0.037
Pooled Norwegian series stages I-III, multivariable Cox model, excluding *KRAS*, *BRAF*^{V600E} and *CDX2* as covariables, in chemotherapy-untreated patients, RCC2, Five-year RFS	S. table 6	-1.2 – 8.1 (-0.19)	0.87 (0.77 – 0.97)	0.012
Norwegian series 2, Chemotherapy-untreated stage I-III patients, interaction between RCC2 and *TP53*, Five-year RFS	S. figure 14	-1.02 – 7.2 (-0.04)	0.82 (0.49 – 1.38)	0.46

Supplementary Methods

MSI scoring by immunohistochemistry. Two 4-plex stains were performed in the Norwegian series 2 against the four mismatch repair (MMR) proteins, MLH1, MSH6, MSH2 and PMS2. Multiplexed fluorescent IHC was performed on 4 µm thick tissue sections by following the Opal protocol (PerkinElmer/Akoya) with a 4-plex kit (NEL810001KT, PerkinElmer/Akoya). The Opal protocol was followed except that slide deparaffinization, antigen retrieval and antibody stripping were all performed using EnVision™ FLEX Target Retrieval Solution 3-in-1, high/low pH, (Agilent/DAKO, codes K8005 and K8004) in the PT-link module (Agilent/DAKO). Staining against MLH1 and MSH6 was performed as follows: initial slide deparaffinization/antigen retrieval in High pH buffer followed by detection of anti-MLH1 (undiluted, Clone ES05, Agilent/DAKO) using Opal 570, antibody stripping in High pH buffer followed by detection of anti-MSH6 (undiluted, Clone EP49, Agilent/DAKO) using Opal 520, antibody stripping in High pH buffer followed by detection of a cocktail of antibodies (epithelial-cocktail) consisting of anti-E-cadherin (1:20.000, Clone 36, BD-Biosciences), anti-pan Cytokeratin (1:4000, C-11, Abcam) and anti-pan Cytokeratin Type I/II (1:2000, AE1/AE3, Thermo Fisher) using Opal 690. After a final antibody stripping procedure in Low pH buffer, nuclei were stained with DAPI and sections were mounted in Prolong™ Diamond Antifade Mountant (Thermo Fisher). The same protocol was followed for MSH2 (undiluted, Clone FE11, Agilent/DAKO; detected with Opal 570) and PMS2 (undiluted, Clone EP51, Agilent/DAKO; detected with Opal 520).

The two stains against the MMR proteins were imaged with the Vectra 3 imaging platform (PerkinElmer/Akoya), visually examined and scored either positive or negative for each of the four proteins. If a sample was negative in the tumor compartment for at least one of the four proteins it was deemed microsatellite instable (MSI). If staining against all proteins was present it was deemed microsatellite stable (MSS). This analysis was done blinded to the MSI status that was available for 339 patients and which were previously assessed by PCR. Of the 798 samples in the Norwegian series 2, 734 were evaluable for MSI status by immunohistochemistry (IHC). Within the 315 samples for which we had overlapping data between methods we found a concordance of 96.5% (data not shown). For patients who were scored into differing categories between methods, the results from PCR were used.

Standard immunohistochemistry in the QUASAR2 cohort. Chromogenic IHC, visualized with the chromogen DAB, was performed using a rabbit polyclonal antibody against RCC2 (Novus Biologicals, Cat. No. NB110-40619), and samples were scored visually according to the Allred method, as previously described.[1] Samples with a cytoplasmic RCC2 Allred score <5 were classified as having low expression of RCC2.

Immunocytochemistry. An RCC2 knockout cell-line was purchased from Horizon Discovery along with its parental HAP1 cell-line. These were grown according to the vendor’s protocol prior to formalin-fixation and paraffin-embedding. Immunocytochemistry on 4 µm thick sections of the FFPE cellular pellets was performed by indirect detection with DAB (3,3’-diaminobenzidine) on an Autostainer Link 48 System (Agilent/DAKO) with a PT-link module. Deparaffinization and antigen retrieval were carried out for 20 minutes at 97°C using EnVision™ FLEX Target Retrieval Solution 3-in-1, high pH, (Agilent/Dako, K8004), in 65°C preheat mode. Subsequent staining was performed with the EnVision FLEX kit (Agilent/DAKO, code K800021-2) together with anti-RCC2 (1:100, monoclonal rabbit, Clone D14F3, Cell Signaling) and FLEX+ linker for rabbit primary antibody (Agilent/DAKO, code K8009). Mayer’s hematoxylin (diluted 1:10, Agilent/DAKO, code S3309) was used as counterstain. Finally, the slides were dehydrated in a graded ethanol series and clarified in xylene before they were mounted onto glass slides using Richard-Allan Scientific Cytoseal mounting media (Thermo Fisher Scientific).

RCC2 staining by multiplexed fluorescent IHC. Multiplexed fluorescent IHC was performed on 4 µm thick tissue sections by following the Opal protocol (PerkinElmer/Akoya) with a 4-plex kit (NEL810001KT, PerkinElmer/Akoya). The Opal protocol was followed except that slide deparaffinization, antigen retrieval and antibody stripping were all performed using EnVision™ FLEX Target Retrieval Solution 3-in-1, high/low pH, (Agilent/DAKO, codes K8005 and K8004) in the PT-link module (Agilent/DAKO). 4-plex staining against RCC2 and Cortactin (data on Cortactin not used in the current study) was performed as follows: initial slide deparaffinization/antigen retrieval in High pH buffer followed by detection of anti-RCC2 (1:100, monoclonal rabbit, Clone D14F3, Cell Signaling) using Opal 570, antibody stripping in High pH buffer followed by detection of anti-Cortactin (1:500, monoclonal mouse, Clone 771716, R&D systems) using Opal 520, antibody stripping in High pH buffer followed by detection of a cocktail of antibodies (epithelial-cocktail) consisting of anti-E-cadherin (1:10000, Clone 36, BD-Biosciences), anti-pan Cytokeratin (1:2000, C-11, Abcam) and anti-pan Cytokeratin Type I/II (1:1000, AE1/AE3, Thermo Fisher) using Opal 690. After a final antibody stripping procedure in Low pH buffer, nuclei were stained with DAPI and sections were mounted in Prolong™ Diamond

Bergsland CH, *et al. ESMO Open* 2020; 5:e001040. doi: 10.1136/esmoopen-2020-001040

Antifade Mountant (Thermo Fisher). Determination of optimal primary antibody concentrations and testing for complete removal of antibodies between sequential rounds of staining was performed on separate test-TMAs prior to staining of the patient series (data not shown). A negative control slide was included during the staining procedures, in which primary antibody was omitted. No signal above noise was detected in the negative control (data not shown).

Digital image analysis, Vectra/Inform system (Oslo University Hospital, OUH). The samples were multispectrally imaged using the Vectra 3 Imaging platform (PerkinElmer/Akoya) at 20x magnification. The resulting multispectral images were then analyzed in Inform software (version 2.3.0, PerkinElmer). Spectra derived from images of samples stained with each fluorophore individually were used to spectrally un-mix the 4-plex images, and tissue auto-fluorescence was removed by using a spectrum derived from unstained tissue. Signal from the epithelial-cocktail and DAPI channels were used to train an algorithm to classify the tissue into epithelial (tumor) and stromal regions. Individual nuclei were segmented using the DAPI signal and cytoplasmic area was constructed by setting the inner distance to nuclei to 1 pixel and the outer distance to 10 pixels. Data obtained with this platform were used to perform all downstream analyses.

Digital image analysis, Zeiss/CellProfiler system (Institute for Molecular Medicine Finland, FIMM). Only the Norwegian series 1 was analyzed on this platform and the data obtained was used only to compare biomarker scores obtained with different instrumental setups. Due to technical differences between the digital image analysis pipelines, data on 39/853 samples scored by the Vectra/Inform system were lacking from the analysis based on the Zeiss/CellProfiler system, leaving 814 overlapping for comparison.

4-channel images were acquired using Metafer 5 scanning and imaging platform (MetaSystems, Germany) consisting of AxioImager.Z2 (Zeiss, Germany) microscope equipped with Zeiss Plan-Apochromat 20x objective (NA 0.8), CoolCube 2m CCD camera (MetaSystems, Germany), PhotoFluor LM-75 (89 North) metal-halide light source, and Zeiss EPLAX VP232-2 power supply. DAPI, FITC, Cy3, Cy5 filters were used with the following exposure times: DAPI = 5.3 ms, FITC (Cortactin) = 5.2 ms, Cy3 (RCC2) = 2.7 ms, Cy5 (epithelial cocktail) = 30 ms. Nine field-of-views were acquired per TMA spot, composed using VSlide (Metasystems), and the images were exported as one tiled image per spot as Lossless compressed TIFFs (95% resolution, 0.4µm/pixel) for image analysis, which was done using CellProfiler (2.2.0). First in the analysis, TMA spots were detected and binarized using following modules: ImageMath(MaxAllChannels); Morph (close disc 60); IdentifyPrimaryObjects (manual thresh 0.1, smoothing 500, no dividing lines, filter min 1.5 million pixels). Then, nuclei were detected using following modules: Enhance dark holes and fill (2-20); IdentifyPrimaryObjects (Adaptive Otsu's, two classes, weighted variance, automatic smoothing, adaptive window size 12, intensity for dividing nuclei with smoothing filter 1, suppress local maxima 14). Cytoplasm objects were generated by expanding nuclei objects by 20 pixels and removing 2 pixels at the nuclear border (between 2 to 20 pixels outside of nuclei). Epithelium was segmented using following modules: IdentifyPrimaryObjects (Adaptive Otsu's, two classes, weighted variance, automatic smoothing, adaptive window size image size, no dividing lines, filter min area 800; ConvertToBinary; Enhance and fill holes 2-20; and Morph (close disc 20). Mean channel intensities were measured within epithelial binary mask either in nuclei or cytoplasm (MaskObjects) using MeasureImageIntensity module, and results were exported as mean values per image (per TMA spot).

Scoring of digitally analyzed RCC2. All digitally analyzed scores were calculated as the mean signal intensity within the cytoplasmic area of the tumor tissue compartment for each patient sample. Dichotomization of the continuous scores was performed within each cohort by setting a threshold based on the proportions of patients in the strong and weak categories of cytoplasmic RCC2 staining originally reported.[1] This cutoff was at the 41st percentile. Continuous RCC2 scores were standardized within each cohort (Norwegian series 1 & 2) by mean-centering and scaling by the standard deviation. The range of continuous RCC2 scores in the NS1 after scaling was from -1.23 to 8.11, and in the NS2 from -1.02 to 7.15.

Bergsland CH, *et al. ESMO Open* 2020; 5:e001040. doi: 10.1136/esmoopen-2020-001040

Supplementary References
1. Bruun J, Kolberg M, Ahlquist TC, et al. Regulator of Chromosome Condensation 2 Identifies High-Risk Patients within Both Major Phenotypes of Colorectal Cancer. *Clin Cancer Res* 2015; **21**(16): 3759–70.
2. McShane LM, Altman DG, Sauerbrei W, et al. Reporting Recommendations for Tumor Marker Prognostic Studies (REMARK). *J Natl Cancer Inst* 2005; **97**(16): 1180–4.
